# The lichens of the Majella National Park (Central Italy): an annotated checklist

**DOI:** 10.3897/mycokeys.78.62362

**Published:** 2021-03-29

**Authors:** Gabriele Gheza, Luca Di Nuzzo, Chiara Vallese, Renato Benesperi, Elisabetta Bianchi, Valter Di Cecco, Luciano Di Martino, Paolo Giordani, Josef Hafellner, Helmut Mayrhofer, Pier Luigi Nimis, Mauro Tretiach, Juri Nascimbene

**Affiliations:** 1 BIOME Lab, Department of Biological, Geological and Environmental Sciences, Alma Mater Studiorum – University of Bologna, Via Irnerio 42, 40126, Bologna, Italy University of Bologna Bologna Italy; 2 Department of Biology, University of Florence, Via La Pira 4, 50121, Florence, Italy University of Florence Florence Italy; 3 Majella National Park, Via Badia 28, 67039, Sulmona, Italy Majella National Park Sulmona Italy; 4 Department of Pharmacy, University of Genova, Viale Cembrano 4, 16148, Genova, Italy University of Genova Genova Italy; 5 Division of Plant Sciences, Institute of Biology, NAWI Graz, University of Graz, Holteigasse 6, 8010, Graz, Austria University of Graz Graz Austria; 6 Department of Life Sciences, University of Trieste, Via L. Giorgieri 10, 34127, Trieste, Italy University of Trieste Trieste Italy

**Keywords:** Abruzzo, arctic-alpine species, biodiversity hotspot, climate change, lichen biota, Mediterranean mountains, steppic species

## Abstract

The botanical exploration of the Majella National Park has a long tradition dating back to the eighteenth century. However, the lichen biota of this area is still poorly investigated. To provide a baseline for future investigations, in this annotated checklist, we summarised all available information on the occurrence of lichens in the Majella National Park, retrieved from previous literature, herbarium material and original data produced by recent research.

The checklist includes 342 infrageneric taxa. However, seven taxa are considered as dubious, thus setting the number of accepted taxa at 335, i.e. 45.8% of those currently known to occur in the Abruzzo Region. This checklist provides a baseline of the lichens known to occur in the Majella National Park, highlighting the potential of this area as a hotspot of lichen biodiversity, especially from a biogeographical point of view as indicated by the occurrence of several arctic-alpine species that form disjunct populations in the summit area of the massif.

## Introduction

The botanical exploration of the Majella National Park has a long tradition dating back to the eighteenth century, which has provided the basis for the compilation of a recent checklist of vascular plants including 2286 infrageneric taxa (Conti et al. 2019). This massif clearly is a hotspot of plant diversity due to the interaction of physical, climatic and biogeographic factors. In particular, the flora of high-elevation habitats consists of many endemic taxa of high phytogeographic relevance.

On the other hand, the lichen biota of this area is still poorly investigated. Historical data are scanty, the main contribution being that by Nimis and Tretiach (1999), who carried out intensive lichen collections along the eastern part of the Italian peninsula. These authors collected several specimens, currently stored in the TSB herbarium, in at least five localities distributed along a steep elevational gradient, from 500 to 2500 m, in the Majella National Park. Ten years later, Cucchi et al. (2009) studied the microtopography of carbonatic rocks, reporting several endolithic taxa. Overall, these collections revealed several interesting species that were either new to the Abruzzo region or indicative of the biogeographic importance of the Majella massif also for lichens. For example, several arctic-alpine lichens occur there in small and disjunct areas at the southernmost limit of their European distribution, the nearest populations being in the Alps (Nimis 2016).

In 2017, a scientific collaboration started between the administration of the Majella National Park (with its botanical office) and the University of Bologna, under the project “Lichen biodiversity in the Majella National Park”, with the aim of contributing to fill this knowledge gap. Besides pure floristic explorations (e.g. Nascimbene et al. 2019), the research project also included ecological investigations, mainly focused on high elevation areas, for example, a lichen survey on the four GLORIA summits (Di Cecco et al. 2019) and along an elevational transect across the whole main ridge of the massif (Di Nuzzo et al. 2021).

To provide a baseline for future investigations, in this annotated checklist, we have summarised all available information on the occurrence of lichens in the Majella National Park, retrieved from previous literature, herbarium material and original data produced by our research. In this checklist, very few lichenicolous fungi are included. To be treated exhaustively, this component would require specific investigations.

## Materials and methods

### Study area

The Majella National Park (MNP) is located in the central Apennines, Italy, and was established in 1995 by National Law 1991, n. 394, to preserve, protect and enhance the high value of the inherent natural, historical and cultural resources of the area. The Park consists mainly of carbonate mountains, separated by valleys and karst high plateaus, with a broad altitudinal range (130–2,793 m a.s.l.). The Majella massif has more than 60 peaks, with half of them rising above 2,000 m and includes the second highest peak in the Apennines, Mount Amaro (2,793 m). From a bioclimatic point of view, the study area is included in the alpine biogeographical region (Cervellini et al. 2020) and the climate corresponds to the subalpine-alpine humid type as far as the lower summit is concerned, whereas the other summits belong to the alpine humid type (Blasi et al. 2005). The Park’s territories are part of the Natura 2000 network. The boundaries coincide with a Special Protection Area (SPA) for the conservation of wild birds (established by the Birds Directive 79/409/EEC). Furthermore, within the Park, there are four Special Areas of Conservation (SAC), established by the Habitats Directive 92/43/EEC (Di Cecco et al. 2020).

### The data

Occurrence data were retrieved from multiple sources, for a total of 1625 records:

1. critical evaluation of literature records (463 records from 10 publications);
2. 217 records stored in on-line available herbaria, mainly from TSB (Herbarium of the University of Trieste);
3. reliable field observations related to our research project (e.g. only in the case of easily-identifiable species) recorded between 2017 and 2019 (100 records);
4. 845 herbarium records (personal herbarium of JN and GG) related to our research project collected between 2017 and 2019.

All these records were georeferenced and stored in a database.

The following abbreviations were used for the sources of occurrence data: C09 (Cucchi et al. 2009), C73 (Cesati 1873), C86 (Coassini Lokar et al. 1986), GG (personal herbarium and field observations by Gabriele Gheza), J74 (Jatta 1874), J11 (Jatta 1909–1911), JN (personal herbarium and field observations by Juri Nascimbene and collaborators), N19 (Nascimbene et al. 2019), NAP (Herbarium of the University of Naples), NT99 (Nimis and Tretiach 1999), R20 (Ravera et al. 2020), RV96 (Recchia and Villa 1996), T15 (Tretiach 2015), TSB (Herbarium of the University of Trieste).

The specimens collected during our project were identified in the laboratory using a dissecting and a compound microscope. Routine chemical spot tests were performed for most specimens. The identification of sterile crustose lichens (e.g. *Lepraria*-species) was based on standardised thin-layer chromatography (TLC), following the protocols of Orange et al. (2001).

Lichen nomenclature, as well as synonymisation of old records, follow ITALIC 6.0 – The information system on Italian Lichens (Nimis and Martellos 2020), which is mainly based on the checklist of the Italian lichens by Nimis (2016). This source was used also for retrieving information on biological traits, ecological requirements and geographic distribution for each taxon.

Taxa are listed alphabetically. For each taxon, the accepted name, all available records, the altitudinal distribution, habitat preference and/or substrate are reported, whenever information is available. A short note (on ecology, distribution and/or taxonomy) is associated with each noteworthy taxon (e.g. taxa which are new to the region and/or of particular biogeographic or conservation importance). Dubious records are reported at the end of the checklist. For each record, a critical note accounting for the “dubious status” is reported.

## Results

### General overview

The checklist includes 342 infrageneric taxa. However, seven taxa are considered as dubious, thus setting the number of accepted taxa at 335, i.e. 45.8% of the those currently known to occur in the Abruzzo Region. In the following, the main traits of the lichen biota are detailed:

1. growth forms: five taxa (1.5%) are leprose, 199 (59.4%) are crustose (161 crustose, 13 placodiomorph, 25 endolithic), 16 (4.9%) are squamulose, 80 (23.9%) are foliose (52 broad-lobed, 25 narrow-lobed, three umbilicate) and 34 (10.2%) are fruticose (31 fruticose, three filamentous). Only four taxa (1.2%) are non-lichenised, lichenicolous fungi: Arthonia galactinaria, Carbonea vitellinaria, Merismatium decolorans and Opegrapha rupestris.
2. photobionts (only lichenised taxa): 296 taxa (88.6%) are chlorolichens (282 with a chlorococcoid photobiont and 16 with a trentepohlioid photobiont), 37 taxa (11.2%) are cyanolichens (35 with a filamentous cyanobacterium and two with a coccaceous cyanobacterium), and one species (0.2%), Peltigera leucophlebia, is a cephalolichen with both a chlorococcoid and a cyanobacterial (in external cephalodia) photobiont.
3. main reproductive strategies: 257 taxa (76.8%) mainly disperse by sexual reproduction, forming ascospores in apothecia or perithecia, while 77 taxa (23.2%) disperse by asexual reproduction (22 by means of isidia or isidia-like structures, 47 by means of soredia or soredia-like structures and seven mainly by means of thallus fragmentation).
4. substrates: 122 taxa (36.6%) are mainly epiphytic, four (1.2%) mainly lignicolous, 126 (38.1%) mainly saxicolous, 78 (22.8%) mainly terricolous and five (1.3%) are lichenicolous on saxicolous lichens: Placocarpus schaereri on Protoparmeliopsis versicolor, Placopyrenium canellum on Circinaria calcarea, Verrucula biatorinaria on Calogaya biatorina, Verrucula coccinearia on Caloplaca coccinea and Verrucula granulosaria on Flavoplaca granulosa. Some species can occur on more than one substrate.

### Noteworthy taxa

The checklist includes several noteworthy taxa, expecially from a biogeographical perspective. Twenty taxa are new to the Abruzzo Region: *Arthrorhaphis citrinella*, *Biatorella hemisphaerica*, *Blastenia ammiospila*, *Blastenia subathallina*, *Calogaya bryochrysion*, *Circinaria hispida*, *Cladonia cariosa*, *Gyalolechia bracteata*, *Heppia adglutinata*, *Myriolecis perpruinosa*, *Peltigera elisabethae*, *Peltigera lepidophora*, *Ramonia luteola*, *Rinodina roscida*, *Rostania ceranisca*, *Scytinium imbricatum*, Solorina bispora subsp. macrospora, *Toninia subnitida*, *Toniniopsis coelestina* and *Trapeliopsis gelatinosa*. Additionally, Scoliciosporum umbrinum var. corticicolum is formally new to Italy (see note below), while *Halecania lecanorina* is formally new to Abruzzo on the basis of an old literature record that is accepted here (see note).

One taxon is known to occur in Italy only for the record reported here (*Thelidium dionantense*), while 16 taxa are the only records for both peninsular and central Italy (*Agonimia gelatinosa*, *Caloplaca cacuminum*, *Circinaria hispida*, *Cladonia polycarpoides*, *Lecidea speirodes*, *Parabagliettoa disjuncta*, *Polyblastia dermatodes*, *Polyblastia verrucosa*, *Rhizocarpon atroflavescens*, *Rinodina roscida*, *Rostania ceranisca*, *Scytinium imbricatum*, Solorina bispora subsp. macrospora, *Thelidium dionantense*, *Toniniopsis coelestina* and *Verrucula coccinearia*).

Twenty-six taxa (*Allocetraria madreporiformis*, *Arthrorhaphis citrinella*, Aspicilia verrucosa var. verrucosa, *Athallia saxifragarum*, *Bilimbia microcarpa*, *Blastenia ammiospila*, *Caloplaca cacuminum*, *Caloplaca stillicidiorum*, *Candelariella commutata*, *Cetraria ericetorum*, *Farnoldia hypocrita*, *Farnoldia micropsis*, *Flavocetraria nivalis*, Lecanora epibryon var. epibryon, *Lecidella wulfenii*, Myriolecis zosterae var. palanderi, *Ochrolechia upsaliensis*, *Ophioparma ventosa*, *Parvoplaca tiroliensis*, *Phaeorrhiza nimbosa*, Physconia muscigena var. muscigena, *Rhizocarpon umbilicatum*, *Rinodina roscida*, *Rostania ceranisca*, *Rusavskia sorediata* and Solorina bispora subsp. bispora) have an arctic-alpine distribution, several of them being at their southernmost distribution limit in Italy, or even in Europe, as in the case of *Allocetraria madreporiformis* and *Caloplaca cacuminum*.

The record of the steppic lichen *Circinaria hispida* provides a connection between the main area of distribution of this taxon (Eastern Europe and Central Asia) and its scattered Western European populations (Northern Italy, Spain).

Finally, thirteen epiphytic taxa are of conservation interest, being included in the Red List of epiphytic lichens of Italy (Nascimbene et al. 2013): *Calogaya lobulata* (VU), *Cetrelia olivetorum* (NT), *Enchylium ligerinum* (NT), *Eopyrenula leucoplaca* (NT), *Gyalecta ulmi* (NT), *Heterodermia speciosa* (NT), *Leptogium hildenbrandii* (NT), *Lobaria pulmonaria* (LC), *Melaspilea enteroleuca* (NT), *Nephroma resupinatum* (NT), *Parmeliella triptophylla* (NT), *Ramonia luteola* (VU) and *Sclerophora pallida* (VU).

## Annotated checklist

***Acarospora cervina* A. Massal.**

Roccacaramanico (NT99); Anticima Femmina Morta (JN: 2017). – From the montane (1000 m: NT99) to the alpine (2420 m: JN) belt. On rock (JN).

***Acarospora glaucocarpa* (Ach.) Körb.**

Valle dell’Orfento (J74). – This record is the only one available from Abruzzo (Nimis 1993, 2016); despite the fact that it was not confirmed by recent exploration, it is considered as reliable, since this is a widespread, common species (Nimis 2016).

***Acarospora macrospora* (Hepp) Bagl.**

M. Focalone, near Bivacco Fusco (NT99). – In the alpine belt (2500 m: NT99). – Previously reported from Abruzzo only by Grillo and Romano (1987) from the Abruzzo National Park.

**Acrocordia conoidea (Fr.) Körb. var. conoidea**

Pretoro, Colle dell’Angelo (NT99); below the Maielletta (TSB: 2005); road between Lettomanoppello and Passo Lanciano (TSB: 2005). – In the montane belt (1080–1350 m: TSB). In a beech forest (TSB). On calcareous rock (TSB).

**Acrocordia gemmata (Ach.) A. Massal. var. gemmata**

Valico della Forchetta (TSB: 1996); Val di Foro (JN: 2018). – From the lower (970 m: JN) to the upper montane belt (1360 m: TSB). On bark of *Fagus* (TSB; JN).

***Agonimia gelatinosa* (Ach.) M. Brand & Diederich**

Trail between Blockhaus and M. Focalone (T15; TSB: 2005); Femmina Morta (JN: 2017). – In the subalpine belt (2300–2420 m: T15; TSB; JN). In calcareous grasslands (T15; TSB; JN). On organic soil (T15; TSB; JN). – These are the only known records for Abruzzo and peninsular Italy and, thus, also the southernmost ones in Italy (Nimis 2016).

***Agonimia tristicula* (Nyl.) Zahlbr.**

Roccacaramanico (NT99); at 20 sites along the main ridge of the Majella massif between 2139 and 2664 m (JN: 2018, 2019). – From the montane (1000 m: NT99) to the alpine (2664 m: JN) belt. In high-altitude open habitats (JN). On soil (JN).

***Allocetraria madreporiformis* (Ach.) Kärnefelt & A. Thell**

M. Amaro (C73; J11); Tavola Rotonda (JN: 2017); Vetta Femmina Morta (JN: 2017); Colle d’Acquaviva (JN: 2017); Anticima M. Acquaviva (JN: 2016); M. Macellaro (JN: 2017, 2018); between Iaccione and Piano Amaro (JN: 2017); Piano Amaro (JN: 2017); Grotta Canosa (JN: 2017); Sella di Grotta Canosa (JN: 2017); between Grotta Canosa and M. Amaro (JN: 2017); M. Amaro (JN: 2017); between M. Acquaviva and M. Focalone (JN: 2017); Cima dell’Altare (JN: 2017); at five sites along the main ridge of Majella between 2322 and 2664 m (JN: 2018, 2019). – From the subalpine (2207 m: JN) to the alpine (2750 m: JN) belt. In high-altitude open habitats (JN). On soil (JN). – These records are the southernmost ones in Europe (cf. Nimis 2016) and confirm the old record by Cesati (1873) from M. Amaro (Jatta 1909–1911, Nimis 1993). According to Nimis (1993), the other old record from Majella by Jatta (1874) from pine bark probably refers to another species. Widespread in the Alps, this species is known from the Apennines only for Abruzzo (Campo Imperatore, in the Gran Sasso massif, Nimis and Tretiach 1999).

***Alyxoria varia* (Pers.) Ertz & Tehler**

Guesthouse of Lama dei Peligni (JN: 2017); Pescocostanzo, Bosco di S. Antonio (N19; JN: 2018). – From the colline (650 m: JN) to the montane (1350 m: JN) belt. On bark of *Acer campestre* (N19; JN), *Fagus* (N19; JN) and *Quercus pubescens* (JN).

***Amandinea punctata* (Hoffm.) Coppins & Scheid.**

Majella (C73); at two sites along the main ridge of Majella between 1825 and 2091 m (JN: 2019). – In the subalpine belt (1825–2091 m: JN). In high-altitude open habitats (JN). On plant debris (JN).

***Anaptychia ciliaris* (L.) A. Massal.**

Majella (C73; J74); Valico della Forchetta (TSB: 1996); Monti Pizzi near S. Domenico (JN: 2017); Lama dei Peligni (JN: 2017); along the highway Strada Statale 164 (JN: 2018); Pescocostanzo, Bosco di S. Antonio (N19; JN: 2018); Valle di Mario (JN: 2018); Piano Cerreto near Campo di Giove (JN: 2018); Cansano (JN: 2018). – From the colline (650 m: JN) to the montane (1434 m: JN) belt. On bark of *Acer campestre* (N19), *Acer pseudoplatanus* (N19; JN), *Fagus* (N19; JN) and *Quercus cerris* (JN).

***Arthonia apatetica* (A. Massal.) Th. Fr.**

Hermitage of M. Morrone (NT99; TSB: 1997). – In the colline belt (500 m: NT99; TSB). On bark of *Fraxinus ornus* (TSB). – This is the only known record for Abruzzo (Nimis 2016).

***Arthonia atra* (Pers.) A. Schneid.**

Pescocostanzo, Bosco di S. Antonio (N19; JN: 2018). – In the montane belt (1350 m: JN). On bark of *Fagus* (N19; JN).

***Arthonia calcarea* (Sm.) Ertz & Diederich**

Pretoro, Colle dell’Angelo (NT99). – In the montane belt (1200 m: NT99). – This is the only known record for Abruzzo (Nimis 2016).

***Arthonia calcicola* Nyl.**

Trail between Lettomanoppello and Passo Lanciano (T15; TSB: 2005). – In the montane belt (1080 m: T15). On calcareous rock (T15). – This is the only known record for Abruzzo (Nimis 2016).

***Arthonia fusca* (A. Massal.) Hepp**

Anticima M. Acquaviva (JN: 2017). – In the alpine belt (2700 m: JN). On calcareous rock (JN).

***Arthonia galactinaria* Leight.**

Roccacaramanico (NT99). – In the montane belt (1000 m: NT99). Parasite on *Myriolecis dispersa* (NT99). – This record was reported under *Arthonia clemens* (Tul.) Th. Fr. by Nimis and Tretiach (1999), but later Nimis (2016) moved it under *A. galactinaria*, since *A. clemens* is recognised to parasitise only species of *Rhizoplaca*.

***Arthonia mediella* Nyl.**

Pescocostanzo, Bosco di S. Antonio (J19; JN: 2018). – In the montane belt (1350 m: JN). On bark of *Acer campestre* (N19; JN) and *Fagus* (N19).

***Arthonia radiata* (Pers.) Ach.**

Pretoro, Colle dell’Angelo (NT99); Valico della Forchetta (TSB: 2016); Pescocostanzo, Bosco di S. Antonio (N19; JN: 2018); along the highway Strada Statale 164 (JN: 2018); Centiata, Villaggio Mirastelle (JN: 2018). – In the montane belt (1200–1420 m: JN). On bark of *Fagus* (N19; JN).

***Arthrorhaphis citrinella* (Ach.) Poelt**

At two sites along the main ridge of Majella between 2582 and 2592 m (JN: 2019). – In the alpine belt (2582–2592 m: JN). In high-altitude open habitats (JN). On soil (JN). – New to Abruzzo. These records are located between the main Italian range of the species on the Alps and the disjunct populations occurring on the highest mountains of Calabria and Sicily (Nimis 2016).

**Aspicilia verrucosa (Ach.) Körb. subsp. verrucosa**

Grotte di Celano near M. Blockhaus (NT99; TSB: 1996); near Bivacco Fusco (JN: 2016); Anticima M. Acquaviva (JN: 2016); Sella di Grotta Canosa (JN: 2017); Anticima Femmina Morta (JN: 2017); Femmina Morta (JN: 2017); at 13 sites along the main ridge of Majella between 1997 and 2664 m (JN: 2018, 2019). – From the submontane (1997 m: JN) to the alpine (2664 m: JN) belt. In high-altitude open habitats (JN). On bryophytes and plant debris (JN).

**Aspicilia verrucosa (Ach.) Körb. subsp. mutabilis (Ach.) Cl. Roux**

Caramanico (TSB). – In the lower montane belt (820 m: TSB). On bark of deciduous *Quercus* sp. (TSB).

***Athallia holocarpa* (Hoffm.) Arup, Frödén & Søchting**

Majella (C73; J74). – On calcareous rock (J74). – The historical records were not confirmed recently, but the record is considered as reliable, since this is a widespread species (Nimis 2016).

***Athallia inconnexa* (Nylander) S.Y. Kondr. & L. Lökös**

Roccacaramanico (NT99; TSB: 1996); near Martellose (JN: 2017). – From the montane (1000 m: NT99; TSB) to the subalpine (2065 m: JN) belt. On calcareous rock (TSB; JN).

***Athallia pyracea* (Ach.) Arup, Frödén & Søchting**

Pescocostanzo, Bosco di S. Antonio (N19; JN: 2018). – In the montane belt (1350 m: JN). On bark of *Fagus* (N19; JN).

***Athallia saxifragarum* (Poelt) Arup, Frödén & Søchting**

M. Focalone near Bivacco Fusco (NT99); Grotte di Celano near M. Blockhaus (NT99); Anticima M. Acquaviva (JN: 2016); Anticima Femmina Morta (JN: 2017); Femmina Morta (JN: 2017); at five sites along the main ridge of Majella between 2322 and 2634 m (JN: 2018, 2019). – From the subalpine (2150 m: NT99) to the alpine (2634 m: JN) belt. On plant debris (JN).

***Bacidia igniarii* (Nyl.) Oxner**

Vallone Grascito (R20). – In the colline belt (568 m: R20). On bark of *Quercus pubescens* (R20). – This record is the only one available from Abruzzo (Nimis and Martellos 2020).

***Bacidia rubella* (Hoffm.) A. Massal.**

Pescocostanzo, Bosco di S. Antonio (N19; JN: 2018). – In the montane belt (1350 m: JN). On bark of *Acer campestre* (N19), *Fagus* (N19; JN).

***Bacidina arnoldiana* (Körb.) V. Wirth & Vězda**

Pretoro, Colle dell’Angelo (NT99; TSB: 1996). – In the montane belt (1200 m: NT99; TSB: 1996). On calcareous rock (NT99; TSB: 1996).

***Bagliettoa calciseda* (DC.) Gueidan & Cl. Roux**

Passo S. Leonardo (C09); Passo Lanciano (C09); Caramanico (C09); Lettomanoppello (C09); M. Blockhaus (C09). – From the colline (570 m: C09) to the subalpine (2170 m: C09) belt. In open shrublands (C09) and pastures (C09). On calcareous rock (C09).

***Bagliettoa marmorea* (Scop.) Gueidan & Cl. Roux**

Majella (C73; J74); Roccacaramanico (NT99); Passo S. Leonardo (C09); Caramanico (C09). – From the colline (570 m: C09) to the montane (1200 m: C09) belt. In pastures (C09) and open shrublands (C09). On calcareous rock (C09).

***Bagliettoa parmigera* (J. Steiner) Vězda & Poelt**

Roccacaramanico (NT99). – In the montane belt (1000 m: NT99).

***Bagliettoa parmigerella* (Zahlbr.) Vězda & Poelt**

Pretoro, Colle dell’Angelo (NT99). – In the montane belt (1200 m: NT99).

***Biatora beckhausii* (Körb.) Tuck.**

Val di Foro (JN: 2018). – In the montane belt (1200 m: JN). On bark of *Fagus* (JN).

***Biatorella hemisphaerica* Anzi**

Anticima M. Acquaviva (JN: 2016). – In the alpine belt (2600 m: JN). In high-altitude open habitats (JN). On soil (JN). – New to Abruzzo. This is the southernmost record in Italy (Nimis 2016).

***Bilimbia lobulata* (Sommerf.) Hafellner & Coppins**

M. Focalone near Bivacco Fusco (NT99); at three sites along the main ridge of Majella between 2073 and 2664 m (JN: 2018, 2019). – From the subalpine (2073 m: JN) to the alpine (2664 m: JN) belt. In high-altitude open habitats (JN). On soil (JN).

***Bilimbia microcarpa* (Th. Fr.) Th. Fr.**

At one site along the main ridge of Majella (JN: 2019). – In the alpine belt (2634 m: JN). In high-altitude open habitats (JN). On soil (JN).

***Bilimbia sabuletorum* (Schreb.) Arnold**

Grotte di Celano near M. Blockhaus (NT99; TSB: 1996). – In the subalpine belt (2150 m: NT99; TSB). On calcareous soil (TSB).

***Blastenia ammiospila* (Ach.) Arup, Søchting & Frödén**

Tavola Rotonda (JN: 2017). – In the alpine belt (2400 m: JN). In high-altitude open habitats (JN). On plant debris with *Ochrolechia androgyna* and *Lecidella wulfenii* (JN). This is a mainly arctic-alpine to boreal-montane, bipolar lichen and the record reported here is the southernmost in Italy. New to Abruzzo.

***Blastenia ferruginea* (Huds.) A. Massal.**

Hermitage of M. Morrone (NT99; TSB: 1997). – In the colline belt (500 m: NT99; TSB). On bark of *Fraxinus ornus* (TSB).

***Blastenia subathallina* (H. Magn.) Arup & Vondrák**

At two sites along the main ridge of Majella between 2025 and 2085 m (JN: 2019). – In the subalpine belt (2025–2028 m: JN). In high-altitude open habitats (JN). On plant debris (JN). – New to Abruzzo. These are the first records from the Apennines and from peninsular Italy (cf. Nimis 2016).

***Blennothallia crispa* (Huds.) Otálora, P.M. Jørg. & Wedin**

Above Bivacco Fusco (JN: 2016); at one site along the main ridge of Majella (JN: 2018). – In the alpine belt (2490–2579 m: JN). In high-altitude open habitats (JN). On soil (JN).

***Bryoplaca sinapisperma* (DC.) Søchting, Frödén & Arup**

Majella (C73); Campo di Giove (J74); at four sites along the main ridge of Majella between 2560 and 2640 m (JN: 2019). – In the alpine belt (2560–2640 m: JN). In high-altitude open habitats (JN). On soil (JN). – These records from the Majella massif are the southernmost in Italy (cf. Nimis 2016).

***Bryoria fuscescens* (Gyeln.) Brodo & D. Hawksw.**

Majella (C73); Bosco di Pacentro (J74). – On bark (J74). – The historical record was not confirmed recently, but it is considered as reliable, since the ecological requirements of this species (Nimis 2016) occur within the study area.

***Buellia griseovirens* (Sm.) Almb.**

Val di Foro (JN: 2018). – In the montane belt (1200 m: JN). On bark of *Fagus* (JN).

***Buellia spuria* (Schaer.) Anzi**

Majella (C73); Campo di Giove (J74). – This is a silicicolous lichen (Nimis 2016) that likely meets its substrate requirements in the Majella massif on flint limestone.

***Calicium salicinum* Pers.**

Pescocostanzo, Bosco di S. Antonio (N19; JN: 2018). – In the montane belt (1350 m: JN). On bark of *Fagus* (N19; JN).

***Calogaya biatorina* (A. Massal.) Arup, Frödén & Søchting**

Roccacaramanico (NT99; TSB: 1996); Anticima Femmina Morta (JN: 2017). – From the montane (1000 m: NT99; TSB) to the alpine (2420 m: JN) belt. On calcareous rock (TSB).

***Calogaya bryochrysion* (Poelt) Vondrák**

Above Bivacco Fusco (JN: 2016). – In the alpine belt (2490: JN). On soil (JN). – New to Abruzzo. This species is currently known from the Alps and this is the southernmost record in Italy (Nimis 2016).

***Calogaya lobulata* (Flörke) Arup, Frödén & Søchting**

Majella (C73). – The historical record was not confirmed recently, but it is considered as reliable since the ecological requirements of this species (Nimis 2016) occur within the study area. This old record is the only one from the Majella massif. The other records from Abruzzo were collected elsewhere (Nimis 2016). The species is included in the Italian Red List of epiphytic lichens as “vulnerable” (Nascimbene et al. 2013).

***Calogaya pusilla* (A. Massal.) Arup, Frödén & Søchting**

Majella (C73; J74). – The historical records were not confirmed recently, but they are considered as reliable, since this is a widespread species (Nimis 2016).

***Calogaya rouxii* (Gaya, Nav.-Ros. & Llimona) – provisionally placed here, ICN Art. 36.1b**

M. Focalone near Bivacco Fusco (NT99; TSB: 1996); Grotte di Celano near M. Blockhaus (NT99; TSB: 1996). – From the subalpine (2150 m: NT99; TSB) to the alpine (2500 m: NT99; TSB) belt. On calcareous rock (TSB). – These records were reported under Caloplaca arnoldii subsp. arnoldii by Nimis and Tretiach (1999), but later Nimis (in Nimis and Martellos 2020) revised the material, which proved to belong to *C. rouxii*.

***Calogaya schistidii* (Anzi) Arup, Frödén & Søchting**

Roccacaramanico (NT99; TSB: 1996). – In the montane belt (1000 m: NT99; TSB). On saxicolous mosses (TSB).

***Caloplaca cacuminum* Poelt**

Grotte di Celano near M. Blockhaus (NT99; TSB: 1996). – In the subalpine belt (1250 m: NT99; TSB). On calcareous rock (TSB). – This is the only known record for Abruzzo and peninsular Italy (Nimis 2016) and the southernmost in Europe (Nimis and Tretiach 1999; Nimis 2016).

***Caloplaca cerina* (Hedw.) Th. Fr. s.lat.**

Roccacaramanico (NT99; TSB: 1996); Valico della Forchetta (TSB: 1996); hermitage of M. Morrone (NT99; TSB: 1997); Lama dei Peligni (JN: 2017); Pescocostanzo, Bosco di S. Antonio (N19; JN: 2018); Campo di Giove, Piano Cerreto (JN: 2018); along the highway Strada Statale 164 (JN: 2018). – From the colline (500 m: NT99; TSB) to the montane (1420 m: JN) belt. On bark of *Fagus* (N19; TSB; JN), *Fraxinus ornus* (TSB), *Quercus cerris* (JN) and *Ulmus minor* (JN). – *Caloplaca cerina* s. str. is an epiphytic species; the record from M. Blockhaus by Nimis and Tretiach (1999) could refer to *C. stillicidiorum* (see) and is not reported here.

***Caloplaca coccinea* (Müll. Arg.) Poelt**

M. Focalone near Bivacco Fusco (NT99; TSB: 1996); Grotte di Celano near M. Blockhaus (NT99; TSB: 1996); above Bivacco Fusco (JN: 2016); M. d’Ugni (JN: 2017); Anticima Femmina Morta (JN: 2017); M. Macellaro (JN: 2018). – From the subalpine (1770 m: JN) to the alpine (2635 m: JN) belt. On calcareous rock (TSB; JN).

***Caloplaca erythrocarpa* (Pers.) Zwackh**

Majella (C73; J74); Roccacaramanico (NT99); Lama dei Peligni (JN: 2019). – From the colline (635 m: JN) to the montane (1000 m: NT99) belt. On concrete (JN).

***Caloplaca haematites* (Chaub.) Zwackh**

Hermitage of M. Morrone (NT99; TSB: 1997). – In the colline belt (500 m: NT99; TSB). On bark of *Fraxinus ornus* (TSB).

**Caloplaca nubigena (Kremp.) Dalla Torre & Sarnth. var. keissleri (Servít) Clauzade & Cl. Roux**

Grotte di Celano near M. Blockhaus (NT99; TSB: 1996). – In the subalpine belt (2150 m: NT99; TSB). On calcareous rock (TSB).

***Caloplaca stillicidiorum* (Vahl) Lynge**

M. Focalone near Bivacco Fusco (NT99; TSB: 1996); Grotte di Celano near M. Blockhaus (NT99; TSB: 1996); Anticima M. Acquaviva (JN: 2016); above Bivacco Fusco (JN: 2016); Sella di Grotta Canosa (JN: 2017); Anticima Femmina Morta (JN: 2017); at 30 sites along the main ridge of Majella between 1958 and 2681 m (JN: 2018, 2019). – From the subalpine (1958 m: JN) to the alpine (2681 m: JN) belt. In high-altitude open habitats (JN). On plant debris (JN) and calcareous soil (TSB).

***Caloplaca teicholyta* (Ach.) J. Steiner**

Lama dei Peligni (JN: 2019). – In the colline belt (635 m: JN). On concrete (JN).

***Candelaria concolor* (Dicks.) Stein**

Majella (C73; J74). – The historical records were not confirmed recently, but they are considered as reliable, since this is a very widespread species (Nimis 2016), which is probably common in the study area at low elevations.

***Candelariella aurella* (Hoffm.) Zahlbr.**

Majella (C73); Roccacaramanico (NT99; TSB: 1996); M. Focalone near Bivacco Fusco (NT99; TSB: 1996); Anticima Femmina Morta (JN: 2017). – From the montane (1000 m: NT99; TSB) to the alpine (2500 m: NT99; TSB) belt. On calcareous rock (TSB).

***Candelariella commutata* Otte & M. Westb.**

At two sites along the main ridge of Majella between 2634 and 2664 m (JN: 2019). – In the alpine belt (2634–2664 m: JN). In high-altitude open habitats (JN). On soil (JN). – This species was previously reported from Abruzzo, as *C. unilocularis*, only from the Gran Sasso massif by Nimis and Tretiach (1999).

***Candelariella faginea* Nimis, Poelt & Puntillo**

Along the Strada Statale 164 (JN: 2018); Pescocostanzo, Bosco di S. Antonio (N19; JN: 2018). – In the montane belt (1350–1420 m: JN) belt. On bark of *Fagus* (N19; JN).

***Candelariella medians* (Nyl.) A.L. Sm.**

Roccacaramanico (NT99; TSB: 1996). – In the montane belt (1000 m: NT99; TSB). On calcareous rock (TSB).

***Candelariella reflexa* (Nyl.) Lettau**

Tocco da Casauria, Osservanza (RV96). – In the colline belt (370 m: RV96). On bark of *Quercus pubescens* (RV96).

***Candelariella vitellina* (Hoffm.) Müll. Arg.**

M. Focalone near Bivacco Fusco (NT99; TSB: 1996). – In the alpine belt (2500 m: NT99; TSB). On decalcified calcareous rock (TSB).

***Candelariella xanthostigma* (Ach.) Lettau**

Valico della Forchetta (TSB: 1996); Valle di Mario (JN: 2018); at three sites along the main ridge of Majella between 1825 and 2350 m (JN: 2019). – From the montane (1360 m: TSB) to the alpine (2350 m: JN) belt. In high-altitude open habitats (JN). On bark of *Acer pseudoplatanus* (JN), *Fagus* (TSB) and on plant debris (JN).

***Carbonea vitellinaria* (Nyl.) Hertel**

M. Focalone near Bivacco Fusco (NT99; TSB: 1996). – In the alpine belt (2500 m: NT99; TSB). On decalcified calcareous rock (TSB). – A lichenicolous fungus growing on *Candelariella vitellina* (Nimis 2016).

***Catapyrenium cinereum* (Pers.) Körb.**

Anticima M. Acquaviva (JN: 2016); Sella di Grotta Canosa (JN: 2017); Grotta Canosa (JN: 2017); Cima dell’Altare (JN: 2017); at 20 sites along the main ridge of Majella between 2001 and 2660 m (JN: 2018, 2019). – From the upper montane (1535 m: JN) to the alpine (2660 m: JN) belt. In high-altitude open habitats (JN). On soil (JN).

***Catapyrenium daedaleum* (Kremp.) Stein**

At two sites along the main ridge of Majella between 2018 and 2119 m (JN: 2019). – In the subalpine belt (2018–2119 m: JN). In high-altitude open habitats (JN). On soil (JN). – This species was previously reported from Abruzzo only from the Gran Sasso massif by Nimis and Tretiach (1999).

***Catillaria lenticularis* (Ach.) Th. Fr.**

Pretoro, Colle dell’Angelo (NT99; TSB: 1996). – In the montane belt (1200 m: NT99; TSB). On calcareous rock (TSB).

***Catillaria nigroclavata* (Nyl.) J. Steiner**

Hermitage of M. Morrone (NT99; TSB: 1997). – In the colline belt (500 m: NT99; TSB). On bark of *Fraxinus ornus* (TSB).

***Cerothallia luteoalba* (Turner) Arup, Frödén & Søchting**

Vallone Grascito (R20). – In the colline belt (564 m: R20). On bark of *Quercus pubescens* (R20). – This record is the only one available from Abruzzo (Nimis and Martellos 2020).

***Cetraria aculeata* (Schreb.) Fr.**

At one site along the main ridge of Majella (JN: 2019). – In the alpine belt (2322 m: JN). In high-altitude open habitats (JN). On soil (JN).

***Cetraria ericetorum* Opiz**

Femmina Morta (J74); M. Rapina (JN: 2017); Iaccione (JN: 2017); near Campo di Giove (JN: 2018); at two sites along the main ridge of Majella between 1995 and 2020 m (JN: 2018, 2019). – From the montane (1250 m: JN) to the alpine (2367 m: JN) belt. In dry grasslands (JN) and high-altitude open habitats (JN). On soil (JN).

**Cetraria islandica (L.) Ach. subsp. islandica**

Majella (C73); M. Amaro (J74; JN: 2017); Femmina Morta (J74; JN: 2017); M. Focalone near Bivacco Fusco (NT99); above Bivacco Fusco (JN: 2016); Anticima M. Acquaviva (JN: 2016); Anfiteatro Murelle (JN: 2017); Guado di Coccia (JN: 2017); Tavola Rotonda (JN: 2017); Valle di Taranta (JN: 2017); Fondo di Femmina Morta (JN: 2017); trail “Sentiero P1” (JN: 2017); M. Macellaro (JN: 2017); between Iaccione and Piano Amaro (JN: 2017); between M. Amaro and Grotta Canosa (JN: 2017); Sella di Grotta Canosa (JN: 2017); Cima dell’Altare (JN: 2017); Valle Cannella (JN: 2017); Rava del Ferro (JN: 2017); M. Focalone (JN: 2017); M. Pescofalcone (JN: 2017); between M. Pescofalcone and M. Rapina (JN: 2017); M. Rapina (JN: 2017); La Carozza (JN: 2017); Cima Murelle (JN: 2017); Bivacco Fusco (JN: 2017); Martellese (JN: 2017); M. Blockhaus (JN: 2017); at 19 sites along the main ridge of Majella between 1847 and 2765 m (JN: 2018, 2019). – From the subalpine (1623 m: JN) to the alpine (2765 m: JN) belt. In high-altitude open habitats (JN). On soil (JN) and plant debris (JN).

***Cetraria muricata* (Ach.) Eckfeldt**

Anticima M. Acquaviva (JN: 2016); near Campo di Giove (JN: 2018). – From the montane (1250 m: JN) to the alpine (2600 m: JN) belt. In a dry grassland (JN). On soil (JN).

***Cetrelia olivetorum* (Nyl.) W. L. Culb. & C. F. Culb.**

M. Morrone, Impianezza (RV96). – In the colline belt (630 m: RV96). On bark of *Quercus pubescens* (RV96). – One of the few confirmed records from central Italy (Nimis 2016). The species is included in the Italian Red List of epiphytic lichens as “near-threatened” (Nascimbene et al. 2013).

***Circinaria calcarea* (L.) A. Nordin, Savić & Tibell**

Majella (J74); Roccacaramanico (NT99; TSB: 1996); hermitage of M. Morrone (NT99; TSB: 1997). – From the colline (500 m: NT99; TSB) to the montane (1000 m: NT99; TSB) belt. On calcareous rock (TSB).

***Circinaria hispida* (Mereschk.) A. Nordin, Savić & Tibell**

At one site along the main ridge of Majella (JN: 2019). – In the subalpine belt (1997 m: JN). In open habitats (JN). On soil (JN). – New to Abruzzo and to peninsular Italy (cf. Nimis 2016). The only other known Italian record is from Alpi Cozie (Piedmont), not far from the only known French locality in the Maritime Alps (Hafellner et al. 2004; Roux et al. 2017). This is a species typical of cold steppes and deserts which occurs in Eastern Europe, Near Asia, Central Asia and North America; it is found also in *Juniperus* steppes of Central Spain and the scattered occurrences in Italy, France and Greece represent natural connections between the two European disjunctions (Hafellner et al. 2004).

***Circinaria hoffmanniana* (S. Ekman & Fröberg ex R. Sant.) A. Nordin**

Roccacaramanico (NT99; TSB: 1996); Anticima Femmina Morta (JN: 2017). – From the montane (1000 m: NT99; TSB) to the alpine (2420 m: JN) belt. On calcareous rock (TSB).

***Circinaria viridescens* (A. Massal.) – provisionally placed here, ICN Art. 36.1b**

Roccacaramanico (NT99; TSB: 1996). – In the montane belt (1000 m: NT99; TSB). On decalcified calcareous rock (TSB).

***Cladonia cariosa* (Ach.) Spreng.**

At one site along the main ridge of Majella (JN: 2019). – In the subalpine belt (1847 m: JN). In open grasslands (JN). On calcareous soil (JN). – New to Abruzzo.

***Cladonia chlorophaea* (Sommerf.) Spreng.**

Pescocostanzo, Bosco di S. Antonio (N19; JN: 2018). – In the montane belt (1350 m: JN). In a beech forest (JN). On bark of *Fagus* (N19; JN).

***Cladonia coniocraea* (Flörke) Spreng.**

Valico della Forchetta (TSB: 1996). – In the montane belt (1360 m: TSB). On dead wood (TSB).

***Cladonia fimbriata* (L.) Fr.**

Pretoro, Colle dell’Angelo (NT99); summit ridge of M. Majella (JN: 2019). – From the montane (1200 m: NT99) to the subalpine (2091 m: JN) belt. On soil (JN).

***Cladonia foliacea* (Huds.) Willd.**

Roccacaramanico (NT99; TSB: 1996); Valle di Fara (JN: 2017); Capo Le Macchie (JN: 2017); Campo di Giove (JN: 2018); Cansano (JN: 2018); Palena (GG: 2018). – From the lower (800 m: JN) to the upper montane (1250 m: JN) belt. In dry grasslands (NT99; JN; GG). On calcareous soil (NT99; JN; GG). – The calciphilous ecotype, which occurs in the study area, has been considered for long as a separate species, *Cladonia convoluta* (Lam.) Anders, but recent studies proved that it belongs to the same species as the acidophilous ecotype (Pino Bodas et al. 2018).

**Cladonia furcata (Huds.) Schrad. subsp. furcata**

Near Campo Giove (JN); at two sites along the main ridge of Majella between 1995 and 2322 m (JN: 2019). – From the montane (1250 m: JN) to the subalpine (2322 m: JN) belt. In dry grasslands (JN) and high-altitude open habitats (JN). On soil (JN).

**Cladonia furcata (Huds.) Schrad. subsp. subrangiformis (L. Scriba ex Sandst.) Pišút**

Roccacaramanico (NT99; TSB: 1996); Capo Le Macchie (JN: 2017). – In the montane belt (875–1000 m: NT99; JN). In calcareous dry grasslands (JN). On calcareous soil (NT99; JN).

***Cladonia humilis* (With.) J.R. Laundon**

Maiellone (C86; NAP: 1872). – This is the only known record for Abruzzo (Nimis 2016).

***Cladonia ochrochlora* Flörke**

Valle dell’Orfento (J74). – On soil (J74).

***Cladonia pocillum* (Ach.) Grognot**

Blockhaus, Grotte di Celano (NT99; TSB: 1996); near Bivacco Fusco (JN: 2016); near Femmina Morta (JN: 2017); Anticima Femmina Morta (JN: 2017); Tavola Rotonda (JN: 2017); trail between Rifugio Pomilio and M. Blockhaus (GG: 2018); at seven sites along the main ridge of Majella between 2025 and 2640 m (JN: 2018, 2019). – From the subalpine (1499 m: JN) to the alpine (2640 m: JN) belt. In open habitats, for example, grasslands (JN; GG). On calcareous soil (TSB; JN; GG).

***Cladonia polycarpoides* Nyl.**

Blockhaus, Grotte di Celano (NT99; TSB: 1996). – In the subalpine belt (2150 m: NT99; TSB). On calcareous soil (NT99; TSB). – This is the only known record for Abruzzo and peninsular Italy (Nimis 2016).

***Cladonia pyxidata* (L.) Hoffm.**

Majella (J74); Campo di Giove (JN: 2017, 2018); at 13 sites along the main ridge of Majella between 1812 and 2645 m (JN: 2018, 2019). – From the montane (1250 m: JN) to the alpine (2645 m: JN) belt. In open habitats, for example, grasslands (JN). On soil (JN).

***Cladonia rangiformis* Hoffm.**

Capo Le Macchie (JN: 2017); trail between Lama dei Peligni and Rifugio Fonte Tarì (JN: 2017); Cansano (JN: 2018); Campo di Giove (JN: 2018). – From the lower (875 m: JN) to the upper montane (1250 m: JN) belt. In dry grasslands (JN). On soil (JN).

***Cladonia symphycarpa* (Flörke) Fr.**

Fara San Martino, Vallone di Santo Spirito (RV96); Anticima Femmina Morta (JN: 2017); Grotta Canosa (JN: 2017); Sella di Grotta Canosa (JN: 2017); Valle di Taranta (JN: 2017); M. Blockhaus (GG: 2018); at 38 sites along the main ridge of Majella between 1847 and 2640 m (JN: 2018, 2019). – From the subalpine (1650 m: JN) to the alpine (2640 m: JN) belt. In calcareous grasslands (JN; GG) and high-altitude open habitats (JN). On calcareous soil (JN; GG).

***Clauzadea metzleri* (Körb.) D. Hawksw.**

Pretoro, Colle dell’Angelo (NT99; TSB: 1996); Roccacaramanico (NT99; TSB: 1996). – In the montane belt (1000–1200 m: NT99; TSB). On calcareous rock (TSB).

***Clauzadea monticola* (Schaer.) Hafellner & Bellem.**

Roccacaramanico (NT99; TSB: 1996). – In the montane belt (1000 m: NT99; TSB). On calcareous rock (TSB). – The only other record of this common species from Abruzzo is from the Gran Sasso massif (Nimis and Tretiach 1999).

***Collema flaccidum* (Ach.) Ach.**

Valico della Forchetta (TSB: 1996); Pescocostanzo, Bosco di S. Antonio (N19; JN: 2016); Val di Foro (JN: 2018). – In the montane belt (1200–1350: JN). On bark of *Acer campestre* (N19) and *Fagus* (N19; TSB; JN).

***Collema furfuraceum* Du Rietz**

Lama dei Peligni (JN: 2017); Pescocostanzo, Bosco di S. Antonio (N19; JN: 2018). – From the colline (650 m: JN) to the montane (1350 m: JN) belt. On bark of *Acer campestre* (N19; JN), *Acer pseudoplatanus* (N19) and *Fagus* (N19; JN).

***Collema nigrescens* (Huds.) DC.**

Caramanico, S. Tommaso (TSB). – In the colline belt (468 m: TSB). On bark of *Quercus* sp. (TSB).

***Collema subflaccidum* Degel.**

Pescocostanzo, Bosco di S. Antonio (N19; JN: 2018). – In the montane belt (1350 m: JN). On bark of *Acer campestre* (N19) and *Fagus* (N19; JN).

***Collema subnigrescens* Degel.**

Lama dei Peligni (JN: 2017); Pescocostanzo, Bosco di S. Antonio (N19; JN: 2016, 2018). – From the colline (650 m: JN) to the montane (1350 m: JN) belt. On bark of *Acer campestre* (N19) and *Fagus* (N19; JN).

***Dacampia hookeri* (Borrer) A. Massal.**

Trail between Blockhaus and M. Focalone (T15; TSB: 2005); Majella, Bivacco Fusco (JN: 2016). – In the subalpine belt (2290–2300 m: T15; TSB; JN). On organic soil (T15; TSB; JN). – This is the only known record for Abruzzo and central Italy (Nimis 2016).

***Dermatocarpon miniatum* (L.) W. Mann**

Majella (C73); ford of S. Antonio (J74); M. Focalone near Bivacco Fusco (NT99); Grotte di Celano near M. Blockhaus (NT99); between Grotta Canosa and M. Amaro (JN: 2017); M. Amaro (JN: 2017). – From the subalpine (2150 m: NT99) to the alpine (2700 m: JN) belt. On rock (JN).

***Diploschistes gypsaceus* (Ach.) Zahlbr.**

Majella (C73; J74). – The historical records were not confirmed recently, but they are considered as reliable, since the ecological conditions required by this species (Nimis 2016) occur within the study area.

***Diplotomma alboatrum* (Hoffm.) Flot.**

Pescocostanzo, Bosco di S. Antonio (N19; JN: 2018). – In the montane belt (1350 m: JN). On bark of *Acer campestre* (N19), *Fagus* (JN).

***Diplotomma hedinii* (H. Magn.) P. Clerc & Cl. Roux**

Grotte di Celano near M. Blockhaus (NT99; TSB: 1996); Anticima Femmina Morta (JN: 2017). – From the subalpine (2150 m: NT99; TSB) to the alpine (2420 m: JN) belt. On calcareous rock (TSB; JN).

***Diplotomma venustum* (Körb.) Körb.**

Roccacaramanico (NT99; TSB: 1996). – In the montane belt (1000 m: NT99; TSB). On calcareous rock (NT99; TSB).

***Enchylium ligerinum* (Hy) Otálora, P.M. Jørg. & Wedin**

Lama dei Peligni (JN: 2017). – In the colline belt (600 m: JN). On bark of *Quercus pubescens* (JN). – The species is included in the Italian Red List of epiphytic lichens as “near-threatened” (Nascimbene et al. 2013).

***Enchylium limosum* (Ach.) Otálora, P.M. Jørg. & Wedin**

Majella (C73); Valle dell’Orfento (J74). – The historical records were not confirmed recently, but they are considered as reliable, since the ecological conditions required by this species (Nimis 2016) occur within the study area.

***Enchylium polycarpon* (Hoffm.) Otálora, P.M. Jørg. & Wedin subsp. polycarpon**

Anticima M. Acquaviva (JN: 2016); Cima dell’Altare (JN: 2017). – From the subalpine (1535 m: JN) to the alpine (2600 m: JN) belt. On calcareous rock (JN).

***Enchylium tenax* (Sw.) Gray**

Majella (C73); Valle dell’Orfento (J74); M. Focalone near Bivacco Fusco (NT99; TSB: 1996); hermitage of M. Morrone (NT99; TSB: 1997); Anticima Femmina Morta (JN: 2017); M. Macellaro (JN: 2018); at 17 sites along the main ridge of Majella between 1812 and 2664 m (JN: 2018, 2019). – From the colline (500 m: NT99; TSB) to the alpine (2664 m: JN) belt. In high-altitude open habitats (JN). On soil (TSB; JN).

***Eopyrenula leucoplaca* (Wallr.) R.C. Harris**

Lama dei Peligni (JN: 2017). – In the colline belt (650 m: JN). On bark of *Quercus pubescens* (JN). – The species is included in the Italian Red List of epiphytic lichens as “near-threatened” (Nascimbene et al. 2013).

***Evernia divaricata* (L.) Ach.**

Ridge beneath Cima Macirenelle (JN: 2020). – In the subalpine belt (1825 m: JN). In a rocky high-altitude habitat (JN). On soil (JN). – This is the only known record for the Majella massif. The species has a scattered distribution on the highest mountains of the Apennines (Nimis 2016) and was reported previously from Abruzzo only by Recchia and Villa (1996).

***Evernia prunastri* (L.) Ach.**

Monti Pizzi near S. Domenico (JN: 2017); Pescocostanzo, Bosco di S. Antonio (N19; JN: 2018); Valle di Mario (JN: 2018); Piano Cerreto near Campo di Giove (JN: 2018); along the Strada Statale 164 (JN: 2018). – In the montane belt (1350–1434 m: JN). On bark of *Acer campestre* (N19), *Acer pseudoplatanus* (JN), *Fagus* (N19; JN) and *Quercus cerris* (JN).

**Farnoldia hypocrita (A. Massal.) Fröberg var. hypocrita**

Anticima M. Acquaviva (JN: 2016). – In the alpine belt (2600 m: JN). On calcareous rock (JN). – Previously reported from Abruzzo only by Jatta (1889).

**Farnoldia jurana (Schaer.) Hertel subsp. jurana**

M. Focalone near Bivacco Fusco (NT99; TSB: 1996); Anticima Femmina Morta (JN: 2017); M. Macellaro (JN: 2018). – In the alpine belt (2420–2635 m: JN). On calcareous rock (TSB; JN).

***Farnoldia micropsis* (A. Massal.) Hertel**

M. Focalone near Bivacco Fusco (NT99; TSB: 1996); Anticima M. Acquaviva (JN: 2017); M. Macellaro (JN: 2018). – In the alpine belt (2500–2700 m: NT99; TSB; JN). On calcareous rock (TSB; JN). – Previously reported from Abruzzo only by Hertel (1967) from the Majella and the Gran Sasso massifs, where it was recorded also by Nimis and Tretiach (1999).

***Flavocetraria nivalis* (L.) Kärnefelt & A. Thell**

Femmina morta (J74; JN: 2017); ridge beneath Cima Macirenelle (JN: 2020). – From the subalpine (1825 m: JN) to the alpine (2408 m: JN) belt. In open habitats (JN). On soil (J74; JN). – These are the only known records for the Majella massif. Common in the Alps, this species occurs only in a few sites of the central Apennines (Nimis 2016); it was previously reported from Abruzzo only from the Gran Sasso massif by Nimis and Tretiach (1999).

***Flavoparmelia caperata* (L.) Hale**

Majella (C73; J74); Pretoro, Colle dell’Angelo (NT99; TSB: 1996). – In the montane belt (1200 m: NT99; TSB). On bark of broadleaved trees (TSB).

***Flavoparmelia soredians* (Nyl.) Hale**

M. Morrone, Impianezza (RV96). – In the colline belt (630 m: RV96). On bark of *Quercus pubescens* (RV96).

***Flavoplaca granulosa* (Müll. Arg.) Arup, Frödén & Søchting**

Roccacaramanico (NT99; TSB: 1996); hermitage of M. Morrone (NT99; TSB: 1997). – From the colline (500 m: NT99; TSB) to the montane (1000 m: NT99; TSB) belt. On calcareous rock (NT99; TSB).

***Gyalecta jenensis* (Batsch) Zahlbr.**

Pretoro, Colle dell’Angelo (NT99; TSB: 1996; S: 1996); below the Majelletta (TSB: 2005). – In the montane belt (1200–1350 m: NT99; TSB). On calcareous rock (TSB).

***Gyalecta ulmi* (Sw.) Zahlbr.**

Pescocostanzo, Bosco di S. Antonio (N19; JN: 2018). – In the montane belt (1350 m: JN). On bark of *Acer campestre* (N19; JN) and *Acer pseudoplatanus* (N19). – The species is included in the Italian Red List of epiphytic lichens as “near-threatened” (Nascimbene et al. 2013).

***Gyalolechia aurea* (Schaer.) A. Massal.**

Majella (C73); Grotte di Celano near M. Blockhaus (NT99; TSB: 1996). – In the subalpine belt (2150 m: NT99; TSB). On soil (TSB). – This species has been previously reported from other localities in the Apennines only from Abruzzo (Gran Sasso massif) by Nimis and Tretiach (1999). The record from M. Blockhaus is the southernmost in Europe (Nimis 2016).

***Gyalolechia bracteata* (Hoffm.) A. Massal.**

Campo di Giove (JN: 2018); at one site along the main ridge of Majella (JN: 2019). – From the montane (1200 m: JN) to the alpine (2634 m: JN) belt. In high-altitude open habitats (JN). On soil (JN). – New to Abruzzo. This is the southernmost record in Italy (Nimis 2016).

**Gyalolechia flavorubescens (Huds.) Søchting, Frödén & Arup var. flavorubescens**

Lama dei Peligni (JN: 2017); Grotta di S. Angelo (JN: 2018). – From the colline (650 m: JN) to the lower montane (850 m: JN) belt. On bark of *Fagus* (JN) and *Quercus pubescens* (JN).

**Gyalolechia flavorubescens (Huds.) Søchting, Frödén & Arup var. quercina (Flagey) Nimis**

Caramanico, S. Tommaso (TSB: 1990). – In the colline belt (468 m: TSB). On bark of *Quercus* sp. (TSB).

***Gyalolechia fulgens* (Sw.) Søchting, Frödén & Arup**

Majella (C73); Valle di Fara (JN: 2017). – In the lower montane belt (800 m: JN). On soil (JN).

***Gyalolechia subbracteata* (Nyl.) Søchting, Frödén & Arup**

Near Bivacco Fusco (JN: 2016); Sella di Grotta Canosa (JN: 2017). – In the alpine belt (2290–2552 m: JN). On soil (JN).

***Halecania lecanorina* (Anzi) M. Mayrhofer & Poelt**

Majella (C73). – This is the only record from Abruzzo, Apennines and peninsular Italy and the southernmost in Italy (Nimis 2016).

***Heppia adglutinata* (Kremp.) A. Massal.**

Femmina Morta (JN: 2019). – In the alpine belt (2330 m: JN). On calciferous soil (JN). New to Abruzzo. This is a cool-temperate to boreal-montane, circumpolar, ephemeral lichen growing in dry, open grasslands.

***Hertelidea botryosa* (Fr.) Printzen & Kantvilas**

Bolognano, Madonna del M. (RV96). – In the colline belt (330 m: RV96). On bark of *Quercus pubescens* (RV96). – This is the only record from Abruzzo and the southernmost for Italy (Nimis 2016).

***Heterodermia speciosa* (Wulfen) Trevis.**

Majella (C73). – The historical record was not confirmed recently, but it is considered as reliable, since the ecological conditions required by this species (Nimis 2016) occur within the study area. It is included in the Italian Red List of epiphytic lichens as “near-threatened” (Nascimbene et al. 2013).

***Heteroplacidium fusculum* (Nyl.) Gueidan & Cl. Roux**

Roccacaramanico (NT99). – In the montane belt (1000 m: NT99). On calcareous rock, lichenicolous on *Circinaria calcarea* (NT99).

***Hyperphyscia adglutinata* (Flörke) H. Mayrhofer & Poelt**

Lama dei Peligni (JN: 2017). – In the colline belt (650 m: JN). On bark of *Ulmus minor* (JN).

***Lathagrium auriforme* (With.) Otálora, P.M. Jørg. & Wedin**

Majella (C73); Caramanico (J74); Roccacaramanico (NT99; TSB: 1996); at three sites in Val di Foro (JN: 2018); below Villaggio Mirastelle (JN: 2018). – From the lower (970 m: JN) to the upper montane (1250 m: JN) belt. On calcareous rock (TSB), terricolous mosses (J74; JN).

***Lathagrium cristatum* (L.) Otálora, P.M. Jørg. & Wedin**

Majella (C73); Caramanico (J74); Roccacaramanico (NT99; TSB: 1996); hermitage of M. Morrone (NT99; TSB: 1996); at one site along the main ridge of Majella (JN: 2019). – From the montane (1000 m: NT99; TSB) to the subalpine (2081 m: JN) belt. In high-altitude open habitats (JN). On calcareous rock (TSB), soil (JN).

***Lathagrium fuscovirens* (With.) Otálora, P.M. Jørg. & Wedin**

Roccacaramanico (NT99; TSB: 1996). – In the montane belt (1000 m: NT99; TSB). On calcareous rock (TSB).

***Lathagrium undulatum* (Flot.) Poetsch**

Majella (C73); at five sites along the main ridge of Majella between 2380 and 2664 m (JN: 2019). – In the alpine belt (2380–2664 m: JN). In high-altitude open habitats (JN). On calcareous rock (JN).

***Lecania cyrtella* (Ach.) Th. Fr.**

Roccacaramanico (NT99; TSB: 1996); at three sites along the main ridge of Majella between 2210 and 2461 m (JN: 2018, 2019). – From the montane (1000 m: NT99; TSB) to the alpine (2461 m: JN) belt. In high-altitude open habitats (JN). On bark of broadleaved trees (TSB) and on small shrubs (JN).

**Lecanora allophana (Ach.) Nyl. f. allophana**

Majella (C73); Pescocostanzo, Bosco di S. Antonio (N19; JN: 2018). – In the montane belt (1350 m: JN). On bark of *Acer campestre* (N19; JN).

***Lecanora argentata* (Ach.) Malme**

Pretoro, Colle dell’Angelo (NT99; TSB: 1996); Valico della Forchetta (TSB: 1996); Monti Pizzi near S. Domenico (JN: 2017). – In the montane belt (1200–1434 m: NT99; TSB; JN). On bark of *Fagus* (TSB; JN).

***Lecanora carpinea* (L.) Vain.**

Roccacaramanico (NT99; TSB: 1996); Pretoro, Colle dell’Angelo (NT99; TSB: 1996); Piano Cerreto near Campo di Giove (JN: 2018); below Villaggio Mirastelle (JN: 2018); along the Strada Statale 164 (JN: 2018). – In the montane belt (1000–1420 m: NT99; TSB; JN). On bark of *Fagus* (TSB; JN), *Quercus cerris* (JN).

**Lecanora chlarotera Nyl. subsp. chlarotera**

Pretoro, Colle dell’Angelo (NT99; TSB: 1996); Lama dei Peligni (JN: 2017); Monti Pizzi near S. Domenico (JN: 2017); Pescocostanzo, Bosco di S. Antonio (N19; JN: 2018); Valle di Mario (JN: 2018); Piano Cerreto near Campo di Giove (JN: 2018); at one site along the main ridge of Majella (JN: 2019). – From the colline (650 m: JN) to the alpine (2350 m: JN) belt. On bark of *Acer pseudoplatanus* (JN), *Fagus* (N19; TSB; JN) and *Quercus cerris* (JN)

**Lecanora epibryon (Ach.) Ach. var. epibryon**

M. Focalone near Bivacco Fusco (NT99; TSB: 1996); Grotte di Celano near M. Blockhaus (NT99; TSB: 1996); near Bivacco Fusco (JN: 2016); Tavola Rotonda (JN: 2017); Femmina Morta (JN: 2017); at one site along the main ridge of Majella (JN: 2019). – From the subalpine (2150 m: NT99; TSB) to the alpine (2634 m: JN) belt. In high-altitude open habitats (JN). On plant debris (TSB; JN).

***Lecanora horiza* (Ach.) Linds.**

Pescocostanzo, Bosco di S. Antonio (N19; JN: 2018). – In the montane belt (1350 m: JN). On bark of *Fagus* (N19; JN).

***Lecanora intumescens* (Rebent.) Rabenh.**

Valico della Forchetta (TSB: 1996); Monti Pizzi near S. Domenico (JN: 2017); Valle di Mario (JN: 2018); Val di Foro (JN: 2018). – From the lower (970 m: JN) to the upper montane (1434 m: JN) belt. On bark of *Fagus* (TSB; JN).

***Lecanora leptyrodes* (Nyl.) Degel.**

Pretoro, Colle dell’Angelo (NT99; TSB: 1996); Lama dei Peligni (JN: 2017); Monti Pizzi near S. Domenico (JN: 2017); Valle di Mario (JN: 2018). – From the lower (650 m: JN) to the upper montane (1434 m: JN) belt. On bark of *Fagus* (TSB; JN).

***Lecanora pulicaris* (Pers.) Ach.**

Grotte di Celano near M. Blockhaus (NT99; TSB: 1996). – In the subalpine belt (2150 m: NT99; TSB). On bark of conifers (TSB).

***Lecanora rouxii* S. Ekman & Tønsberg**

Hermitage of M. Morrone (NT99; TSB: 1997). – In the colline belt (500 m: NT99; TSB). On calcareous soil (TSB). – This is the only known record for Abruzzo and the southernmost in Italy (Nimis 2016).

***Lecanora subcarpinea* Szatala**

Roccacaramanico (NT99; TSB: 1996); Valico della Forchetta (TSB: 1996); Pescocostanzo, Bosco di S. Antonio (N19; JN: 2018). – In the montane belt (1000–1360 m: NT99; TSB). On bark of *Fagus* (N19; TSB; JN).

***Lecanora varia* (Hoffm.) Ach.**

Majella (C73; J74). This old record was not confirmed by recent surveys, but it can be considered reliable since this cool-temperate to circumboreal-montane lichen is common on hard lignum in upland areas, including Mediterranean mountains (Nimis 2016).

***Lecidea berengeriana* (A. Massal.) Nyl.**

M. Focalone near Bivacco Fusco (NT99; TSB: 1996). – In the alpine belt (2500 m: NT99; TSB). On bryophytes and plant debris (TSB).

***Lecidea confluens* (Weber) Ach.**

Majella (C73); Monte Amaro (J74). – This is a silicicolous lichen (Nimis 2016) that likely meets its substrate requirements in the Majella massif on flint limestone.

***Lecidea speirodes* Nyl.**

Grotte di Celano near M. Blockhaus (NT99; TSB: 1996). – In the subalpine belt (2150 m: NT99; TSB). On calcareous rock (TSB). – This is the only known record for Abruzzo and peninsular Italy and the southernmost in Europe (Nimis 2016).

***Lecidella carpathica* Körb.**

Roccacaramanico (NT99; TSB: 1996). – In the montane belt (1000 m: NT99; TSB). On calcareous rock (TSB).

**Lecidella elaeochroma (Ach.) M. Choisy var. elaeochroma f. elaeochroma**

Majella (C73); Roccacaramanico (NT99; TSB: 1996); Pretoro, Colle dell’Angelo (NT99; TSB: 1996); Grotte di Celano near M. Blockhaus (NT99; TSB: 1996); Valico della Forchetta (TSB: 1996); hermitage of M. Morrone (NT99; TSB 1997); Lama dei Peligni (JN: 2017); Monti Pizzi near S. Domenico (JN: 2017); Pescocostanzo, Bosco di S. Antonio (N19; JN: 2018); Valle di Mario (JN: 2018); Piano Cerreto near Campo di Giove (JN: 2018); below Villaggio Mirastelle (JN: 2018); along Strada Statale 164 (JN: 2018); at 9 sites along the main ridge of Majella between 2001 and 2533 m (JN: 2018, 2019). – From the colline (500 m: NT99; TSB) to the alpine (2533 m: JN) belt. In high-altitude open habitats (JN). On bark of *Acer pseudoplatanus* (JN), *Fagus* (N19; TSB; JN), *Fraxinus ornus* (TSB), *Quercus cerris* (JN) and on plant debris (JN).

***Lecidella euphorea* (Flörke) Hertel**

At twenty sites along the main ridge of Majella between 1812 and 2350 m (JN: 2019). – From the subalpine (1812 m: JN) to the alpine (2350 m: JN) belt. In high-altitude open habitats (JN). On *Juniperus* twigs (JN). – It was previously reported from Abruzzo only by Grillo and Romano (1987) from the Abruzzo National Park.

***Lecidella patavina* (A. Massal.) Knoph & Leuckert**

Pretoro, Colle dell’Angelo (NT99; TSB: 1996); M. Focalone near Bivacco Fusco (NT99; TSB: 1996); Grotte di Celano near M. Blockhaus (NT99; TSB: 1996); Anticima Femmina Morta (JN: 2017); Anticima M. Acquaviva (JN: 2017); M. Macellaro (JN: 2018). – From the montane (1200 m: NT99; TSB) to the alpine (2700 m: JN) belt. On calcareous rock (TSB; JN).

***Lecidella wulfenii* (Hepp) Körb.**

Tavola Rotonda (JN: 2017); at one site along the main ridge of Majella (JN: 2019). – In the alpine belt (2322–2398 m: JN). In high-altitude open habitats (JN). On plant debris (JN). – This species was previously reported from Abruzzo only from the Gran Sasso massif by Nimis and Tretiach (1999).

***Lepra albescens* (Huds.) Hafellner**

Lama dei Peligni (JN: 2017); Monti Pizzi near S. Domenico (JN: 2017); Grotta di S. Angelo (JN: 2018); Pescocostanzo, Bosco di S. Antonio (N19; JN: 2018). – From the colline (650 m: JN) to the montane (1440 m: JN) belt. On bark of *Fagus* (N19; JN), *Quercus cerris* (N19) and *Quercus pubescens* (JN).

***Lepraria eburnea* J.R. Laundon**

At one site along the main ridge of Majella (JN: 2019). – In the alpine belt (2573 m: JN). In high-altitude open habitats (JN). On soil (JN).

***Lepraria nivalis* J.R. Laundon**

Lettomanoppello, Fontana del Papa (TSB: 2005). – In the colline belt (500 m: TSB). On calcareous rock (TSB).

***Lepraria vouauxii* (Hue) R.C. Harris**

Anticima M. Acquaviva (JN: 2016). – In the alpine belt (2600 m: JN). On soil (JN).

***Leproplaca xantholyta* (Nyl.) Hue**

Pretoro, Colle dell’Angelo (NT99; TSB: 1996); Valle di Fara (JN: 2017). – In the montane belt (800–1200 m: NT99; TSB; JN). On calcareous rock (TSB; JN).

***Leptogium hildenbrandii* (Garov.) Nyl.**

Majella (C73); Piano dei Mulini (J74); Lama dei Peligni (JN: 2017). – In the colline belt (650 m: JN). On bark of *Quercus pubescens* (JN). – The species is included in the Italian Red List of epiphytic lichens as “near-threatened” (Nascimbene et al. 2013).

***Leptogium saturninum* (Dicks.) Nyl.**

Pescocostanzo, Bosco di S. Antonio (N19; JN: 2016, 2018); Valle di Mario (JN: 2018); at one site along the main ridge of Majella (JN: 2018). – In montane belt (1350 m: JN). In beech-dominatedforests (JN). On bark of *Acer pseudoplatanus* (JN) and *Fagus* (N19; JN).

***Lobaria pulmonaria* (L.) Hoffm.**

Pescocostanzo, Bosco di S. Antonio (N19; JN: 2016, 2018). – In the montane belt (1350 m: JN). On bark of *Fagus* (N19; JN) and *Quercus cerris* (N19). – The species is included in the Italian Red List of epiphytic lichens as “least concern” (Nascimbene et al. 2013).

***Lobothallia controversa* Cl. Roux & A. Nordin**

Majella (C73). This old record was not confirmed by recent survey, but it seems reliable since this is a mainly southern species in Europe, found on hard rocks with optimum in the montane belt (Nimis 2016).

***Lobothallia radiosa* (Hoffm.) Hafellner**

Majella (C73); Valle dell’Orfento (J74); Roccacaramanico (NT99; TSB: 1996). – In the montane belt (1000 m: NT99; TSB). On calcareous rock (TSB).

***Melanelixia glabra* (Schaer.) O. Blanco, A. Crespo, Divakar, Essl., D. Hawksw. & Lumbsch**

Roccacaramanico (NT99; TSB: 1996); Lama dei Peligni (JN: 2017); Pescocostanzo, Bosco di S. Antonio (N19; JN: 2018). – From the colline (650 m: JN) to the montane (1350 m: JN) belt. On bark of *Acer campestre* (N19), *Fagus* (N19; JN) and *Ulmus minor* (JN).

***Melanelixia glabratula* (Lamy) Sandler & Arup**

Valico della Forchetta (TSB: 1996); Lama dei Peligni (JN: 2017); Pescocostanzo, Bosco di S. Antonio (N19; JN: 2018); Valle di Mario (JN: 2018); Piano Cerreto near Campo di Giove (JN: 2018); below Villaggio Mirastelle (JN: 2018); along the highway Strada Statale 164 (JN: 2018). – From the colline (650 m: JN) to the montane (1420 m: JN) belt. On bark of *Acer campestre* (N19), *Acer pseudoplatanus* (JN), *Fagus* (N19; TSB; JN), *Quercus cerris* (JN) and *Ulmus minor* (JN).

***Melanelixia subargentifera* (Nyl.) O. Blanco, A. Crespo, Divakar, Essl., D. Hawksw. & Lumbsch**

Lama dei Peligni (JN: 2017); Pescocostanzo, Bosco di S. Antonio (N19; JN: 2018). – From the colline (650 m: JN) to the montane (1350 m: JN) belt. On bark of *Acer campestre* (N19; JN), *Fagus* (N19; JN) and *Ulmus minor* (JN).

***Melanelixia subaurifera* (Nyl.) O. Blanco, A. Crespo, Divakar, Essl., D. Hawksw. & Lumbsch**

Bosco di S. Antonio (RV96); San Domenico, Monti Pizzi (JN: 2017); Valle di Mario (JN: 2018); along the highway Strada Statale 164 (JN: 2018). – In the montane belt (1340–1434 m: RV96; JN). On bark of *Acer pseudoplatanus* (JN) and *Fagus* (RV96; JN).

***Melanohalea elegantula* (Zahlbr.) O. Blanco, A. Crespo, Divakar, Essl., D. Hawksw. & Lumbsch**

Lama dei Peligni (JN: 2017); Pescocostanzo, Bosco di S. Antonio (N19; JN: 2018). – From the colline (650 m: JN) to the montane (1350 m: JN) belt. On bark of *Acer campestre* (N19), *Fagus* (N19; JN) and *Quercus pubescens* (JN).

***Melanohalea exasperata* (De Not.) O. Blanco, A. Crespo, Divakar, Essl., D. Hawksw. & Lumbsch**

Roccacaramanico (NT99; TSB: 1996); Pretoro, Colle dell’Angelo (NT99; TSB: 1996); Piano Cerreto near Campo di Giove (JN: 2018). – In the montane belt (1000–1200 m: NT99; TSB). On bark of *Quercus cerris* (JN).

***Melaspilea enteroleuca* (Ach.) Ertz & Diederich**

Guesthouse of Lama dei Peligni (JN: 2017). – In the colline belt (650 m: JN). On bark of *Quercus pubescens* (JN). – The species is included in the Italian Red List of epiphytic lichens as “near-threatened” (Nascimbene et al. 2013).

***Merismatium decolorans* (Rehm) Triebel**

Anticima Femmina Morta (JN: 2017). – In the alpine belt (2420 m: JN). In open high-altitude habitat (JN). – A lichenicolous fungus growing on *Cladonia symphycarpa* (JN).

**Micarea lignaria (Ach.) Hedl. var. lignaria**

Bosco di Pacentro (J74). – On bark of *Fagus* (J74). – The historical record was not confirmed recently, but it is considered as reliable, since the ecological conditions required by this species (Nimis 2016) occur within the study area.

***Mycobilimbia pilularis* (Körb.) Hafellner & Türk**

Majella, S. Antonino (J74). – On mosses (J74). – The historical record was not confirmed recently, but is considered as reliable, since the ecological conditions required by this species (Nimis 2016) occur within the study area.

**Myriolecis agardhiana (Ach.) Sliwa, Zhao Xin & Lumbsch subsp. agardhiana**

M. Focalone near Bivacco Fusco (NT99; TSB: 1996); Anticima Femmina Morta (JN: 2017); Anticima M. Acquaviva (JN: 2017); M. Macellaro (JN: 2018). – In the alpine belt (2420–2700 m: JN). On calcareous rock (TSB; JN).

**Myriolecis agardhiana (Ach.) Sliwa, Zhao Xin & Lumbsch subsp. sapaudica (Cl. Roux) Nimis & Cl. Roux**

Grotte di Celano near M. Blockhaus (NT99; TSB: 1996); Anticima Femmina Morta (JN: 2017). – From the subalpine (2150 m: NT99; TSB) to the alpine (2420 m: JN) belt. On calcareous rock (TSB; JN). – The only other record of this taxon from Abruzzo is from the Gran Sasso massif (Nimis and Tretiach 1999). Those from Majella are the southernmost records for Italy (Nimis 2016).

***Myriolecis dispersa* (Pers.) Sliwa, Zhao Xin & Lumbsch**

Majella (C73); Roccacaramanico (NT99). – In the montane belt (1000 m: NT99).

***Myriolecis hagenii* (Ach.) Sliwa, Zhao Xin & Lumbsch**

Popoli, Impianezza (RV96); Pescocostanzo, Bosco di S. Antonio (N19; JN: 2018); Campo di Giove, Piano Cerreto (JN: 2018). – From the colline (630 m: RV96) to the montane (1350 m: JN) belt. On bark of *Acer campestre* (N19), *Fagus* (N19; JN), *Quercus cerris* (JN) and *Quercus pubescens* (RV96).

***Myriolecis perpruinosa* (Fröberg) Sliwa, Zhao Xin & Lumbsch**

Anticima Femmina Morta (JN: 2017). – In the alpine belt (2420 m: JN). On calcareous rock (JN). – New to Abruzzo. This is the southernmost record of the species in Italy (Nimis 2016).

***Myriolecis reuteri* (Schaer.) Sliwa, Zhao Xin & Lumbsch**

M. Focalone near Bivacco Fusco (NT99; TSB: 1996); Grotte di Celano near M. Blockhaus (NT99; TSB: 1996); M. d’Ugni (JN: 2017). – From the subalpine (1770 m: JN) to the alpine (2500 m: NT99; TSB) belt. On calcareous rock (TSB; JN).

***Myriolecis semipallida* (H. Magn.) Sliwa, Zhao Xin & Lumbsch**

Majella (C73); M. Focalone near Bivacco Fusco (NT99; TSB: 1996); Grotte di Celano near M. Blockhaus (NT99; TSB: 1996); Anticima M. Acquaviva (JN: 2017). – From the subalpine (2150 m: NT99; TSB) to the alpine (2700 m: JN) belt. On calcareous rock (TSB; JN).

**Myriolecis zosterae (Ach.) Śliwa, Zhao Xin & Lumbsch var. palanderi (Vain.) Śliwa**

M. Focalone near Bivacco Fusco (NT99); Bivacco Fusco (JN: 2016); Anticima M. Acquaviva (JN: 2016); Anticima Femmina Morta (JN: 2017); Sella di Grotta Canosa (JN: 2017); at 11 sites along the main ridge of Majella between 1997 and 2681 m (JN: 2018, 2019). – From the subalpine (1997 m: JN) to the alpine (2681 m: JN) belt. In high-altitude open habitats (JN). On plant debris (JN).

***Nephroma resupinatum* (L.) Ach.**

Pescocostanzo, Bosco di S. Antonio (N19; JN: 2016, 2018); Monti Pizzi, Valle del Sole (JN: 2017). – In the montane belt (1350–1455 m: JN). On bark of *Fagus* (N19; JN). – The species is included in the Italian Red List of epiphytic lichens as “near-threatened” (Nascimbene et al. 2013).

***Ochrolechia arborea* (Kreyer) Almb.**

Majella (J74). – On bark of *Fagus* (J74). – The historical record was not confirmed recently, but it is considered as reliable, since this is a widespread species (Nimis 2016).

***Ochrolechia pallescens* (L.) A. Massal.**

Majella (C73); Monti Pizzi near S. Domenico (JN: 2017); Pescocostanzo, Bosco di S. Antonio (N19; JN: 2018). – In the montane belt (1350–1434 m: JN). On bark of *Fagus* (N19; J74) and *Quercus cerris* (N19).

***Ochrolechia upsaliensis* (L.) A. Massal.**

Tavola Rotonda (JN: 2017). – In the alpine belt (2398 m: JN). On soil (JN). – The only other record of this taxon from Abruzzo is from the Gran Sasso massif (Nimis and Tretiach 1999). Those from Majella are the southernmost records of this taxon for Italy (Nimis 2016).

***Opegrapha rupestris* Pers.**

Majella (C73; J74). – The historical records were not confirmed recently, but they are considered as reliable, since this is a widespread species. Lichenicolous in various verrucarialean crustose lichens (Nimis 2016).

***Ophioparma ventosa* (L.) Norman**

Majella (C73); Campo di Giove (J74). – This is a silicicolous, arctic-alpine circumpolar lichen (Nimis 2016) that likely meets its substrate requirements in the Majella massif on flint limestone. The southernmost records in Italy are those of Calabria (Nimis 2016).

***Parabagliettoa disjuncta* (Arnold) Krzewicka**

Grotte di Celano near M. Blockhaus (NT99; TSB: 1996). – In the subalpine belt (2150 m: NT99; TSB). On calcareous rock (TSB). – This is the only known record of the species from Abruzzo, peninsular Italy and the Apennines and the southernmost in Italy (Nimis 2016).

***Parabagliettoa dufourii* (DC.) Gueidan & Cl. Roux**

M. Focalone near Bivacco Fusco (NT99; TSB: 1996). – In the alpine belt (2500 m: NT99; TSB). On calcareous rock (TSB). – This is the only known record of the species from Abruzzo (Nimis 2016).

***Parmelia saxatilis* (L.) Ach.**

Majella (C73); S. Antonio (J74). – The historical records were not confirmed recently, but they are considered as reliable, since this is a widespread species (Nimis 2016). However, different cryptic species may occur in the group of *P. saxatilis* (e.g. Molina et al. 2004) and further research is required to clarify which of them occurs in the study area.

***Parmelia submontana* Hale**

Monti Pizzi near S. Domenico (JN: 2017); below Villaggio Mirastelle (JN: 2018); along the highway Strada Statale 164 (JN: 2018). – In the montane belt (1230–1434 m: JN). On bark of *Fagus* (JN).

***Parmelia sulcata* Taylor**

Pretoro, Colle dell’Angelo (NT99; TSB: 1996); Valico della Forchetta (TSB: 1996); Pescocostanzo (TSB); Monti Pizzi near S. Domenico (JN: 2017); Pescocostanzo, Bosco di S. Antonio (N19; JN: 2018); Valle di Mario (JN: 2018); Piano Cerreto near Campo di Giove (JN: 2018); below Villaggio Mirastelle (JN: 2018); along the highway Strada Statale 164 (JN: 2018). – In the montane belt (1200–1434 m: NT99; TSB; JN). On bark of *Acer campestre* (N19), *Acer pseudoplatanus* (N19; JN), *Fagus* (N19; TSB; JN) and *Quercus cerris* (N19; JN).

***Parmeliella triptophylla* (Ach.) Müll. Arg.**

Majella (C73). – The historical record was not confirmed recently, but it is considered as reliable, since the ecological conditions required by this species (Nimis 2016) occur within the study area. It is included in the Italian Red List of epiphytic lichens as “near-threatened” (Nascimbene et al. 2013).

***Parmelina carporrhizans* (Taylor) Poelt & Vězda**

Cerro, Popoli (RV96). – In the colline belt (350 m: RV96). On bark of *Quercus pubescens* (RV96). – This is the only known record from Abruzzi (Nimis 2016).

***Parmelina pastillifera* (Harm.) Hale**

Monti Pizzi near S. Domenico (JN: 2017); Grotta di S. Angelo near Lama dei Peligni (JN: 2018). – From the lower (850 m: JN) to the upper montane (1440 m: JN) belt. On bark of *Fagus* (JN).

***Parmelina quercina* (Willd.) Hale**

Valico della Forchetta (TSB: 1996); Lama dei Peligni (JN: 2017); Piano Cerreto near Campo di Giove (JN: 2018). – From the colline (650 m: JN) to the montane (1350 m: TSB) belt. On bark of *Fagus* (TSB), *Quercus cerris* (JN) and *Ulmus minor* (JN).

***Parmelina tiliacea* (Hoffm.) Hale**

Majella (J74); Lama dei Peligni (JN: 2017); Monti Pizzi near S. Domenico (JN: 2017); Grotta di S. Angelo near Lama dei Peligni (JN: 2018); Valle di Mario (JN: 2018); Pescocostanzo, Bosco di S. Antonio (N19; JN: 2018). – From the colline (650 m: JN) to the montane (1434 m: JN) belt. On bark of *Acer campestre* (N19), *Acer pseudoplatanus* (N19; JN), *Fagus* (N19; JN), *Quercus cerris* (N19) and *Quercus pubescens* (JN).

***Parmotrema perlatum* (Huds.) M. Choisy**

Majella (C73; J74). – The historical records were not confirmed recently, but they are considered as reliable, since this is a widespread species (Nimis 2016).

***Parvoplaca tiroliensis* (Zahlbr.) Arup, Søchting & Frödén**

Grotte di Celano near M. Blockhaus (NT99; TSB: 1996); near Bivacco Fusco (JN: 2016); Anticima M. Acquaviva (JN: 2016); Anticima Femmina Morta (JN: 2017); at 11 sites along the main ridge of Majella between 2322 and 2664 m (JN: 2018, 2019). – From the subalpine (2150 m: NT99; TSB) to the alpine (2664 m: JN) belt. In high-altitude open habitats (JN). On plant debris (TSB; JN). – The only other record of this taxon from Abruzzo is from the Gran Sasso massif (Nimis and Tretiach 1999). Those from Majella are the southernmost records for Italy (Nimis 2016).

***Peccania coralloides* (A. Massal.) Arnold**

Guado di S. Antonio (J74). – On rock (J74). – The historical record was not confirmed recently, but it is considered as reliable, since this is a widespread species (Nimis 2016).

***Peltigera canina* (L.) Willd.**

Majella (C73; J74). – The historical record was not confirmed recently, but it is considered as reliable, since this is a widespread species (Nimis 2016).

***Peltigera collina* (Ach.) Schrad.**

Pescocostanzo (TSB); Pescocostanzo, Bosco di S. Antonio (N19; JN: 2018). – In the montane belt (1350 m: JN). On bark of *Fagus* (N19; TSB; JN).

***Peltigera elisabethae* Gyeln.**

M. Rapina (JN: 2017); Val di Foro (JN: 2018). – From the montane (970 m: JN) to the subalpine (1920 m: JN) belt. On soil (JN) and terricolous mosses (JN). – New to Abruzzo.

***Peltigera horizontalis* (Hudson) Baumg.**

Pescocostanzo, Bosco di S. Antonio (N19; JN: 2016); near Campo di Giove (JN: 2018); Val di Foro (JN: 2018). – In the montane belt (1200–1450 m: JN). In beech woods (JN). On soil (JN), terricolous mosses (JN) and epiphytic mosses on *Fagus* (N19; JN).

***Peltigera lepidophora* (Vain.) Bitter**

Above Bivacco Fusco (JN: 2016). – In the alpine belt (2490 m: JN). On soil (JN). – New to Abruzzo.

***Peltigera leucophlebia* (Nyl.) Gyeln.**

Fara San Martino, Vallone di Santo Spirito (RV96); Blockhaus, Grotte di Celano (NT99). – From the montane (1100 m: RV96) to the subalpine (2150 m: NT99) belt. On soil above calcareous rock (RV96). – These are the only known records for Abruzzo (Nimis 2016).

***Peltigera neckeri* Müll.Arg.**

Valle dell’Orfento (RV96); near Passo Lanciano (JN: 2017); Val di Foro (JN: 2018); at one site along the main ridge of Majella (JN: 2019). – From the colline (530 m: RV96) to the subalpine (2119 m: JN) belt. In high-altitude open habitats (JN). On soil (JN), mosses (RV96).

***Peltigera polydactylon* (Neck.) Hoffm.**

Above Bivacco Fusco (JN: 2016). – In the alpine belt (2490 m: JN). On soil (JN).

***Peltigera praetextata* (Sommerf.) Zopf**

Pescocostanzo, Bosco di S. Antonio (N19; JN: 2016, 2018); Monti Pizzi near S. Domenico (JN: 2017); near Campo di Giove (JN: 2018); Val di Foro (JN: 2018); below Villaggio Mirastelle (JN: 2018). – In the montane belt (970–1450 m: JN). In beech woods (JN). On soil (JN), terricolous mosses (JN), epiphytic mosses on *Fagus* (JN) and bark of *Fagus* (N19; JN).

***Peltigera rufescens* (Weiss) Humb.**

M. Focalone near Bivacco Fusco (NT99; TSB: 1996); near Bivacco Fusco (JN: 2016); Anticima M. Acquaviva (JN: 2016); Campo di Giove (JN: 2017); Colle d’Acquaviva (JN: 2017); Sella di Grotta Canosa (JN: 2017); Campo di Giove (JN: 2018); near Campo di Giove (JN: 2018); trail between Rifugio Pomilio and M. Blockhaus (GG); M. Blockhaus (GG); at nine sites along the main ridge of Majella between 2149 and 2637 m (JN: 2018, 2019). – From the subalpine (1715 m: JN) to the alpine (2637 m: JN) belt. In dry grasslands (JN; GG) and high-altitude open habitats (JN). On soil (TSB; JN; GG).

***Pertusaria coronata* (Ach.) Th. Fr.**

Pescocostanzo, Bosco di S. Antonio (N19; JN: 2018). – In the montane belt (1350 m: JN). On bark of *Fagus* (N19; JN).

***Petractis clausa* (Hoffm.) Kremp.**

Roccacaramanico (NT99; TSB: 1996); below the Maielletta (TSB: 2005). – In the montane belt (1000 m: NT99; TSB). On calcareous rock (TSB). – These records from Majella are the only recent ones from Abruzzo, the others date back to the 19^th^ Century (see literature cited by Nimis 1993).

***Phaeophyscia ciliata* (Hoffm.) Moberg**

Roccacaramanico (NT99; TSB: 1996); Lama dei Peligni (JN: 2017). – From the colline (650 m: JN) to the montane (1000 m: NT99; TSB) belt. On bark of *Ulmus minor* (JN).

***Phaeophyscia orbicularis* (Neck.) Moberg**

Majella (C73); S. Antonio (J74); Caramanico S. Tommaso (TSB: 1990); Roccacaramanico (NT99; TSB: 1996); Lama dei Peligni (JN: 2017); Pescocostanzo, Bosco di S. Antonio (N19; JN: 2018); Piano Cerreto near Campo di Giove (JN: 2018). – From the colline (468 m: TSB) to the montane (1350 m: JN) belt. On bark of *Acer campestre* (JN), *Acer pseudoplatanus* (N19), *Fagus* (N19), *Quercus cerris* (JN) and *Quercus* sp. (TSB).

***Phaeophyscia sciastra* (Ach.) Moberg**

Roccacaramanico (NT99; TSB: 1996). – In the montane belt (1000 m: NT99; TSB). On calcareous rock (TSB).

***Phaeorrhiza nimbosa* (Fr.) H. Mayrhofer & Poelt**

M. Focalone near Bivacco Fusco (NT99; TSB: 1996; JN: 2016). – In the alpine belt (2490–2500 m: NT99; TSB; JN). On calcareous soil (NT99; TSB; JN). – The only other records from Abruzzo are from the Gran Sasso massif (Nimis and Tretiach 1999). Those from Majella are the southernmost records for Italy (Nimis 2016).

***Phlyctis argena* (Spreng.) Flot.**

Lama dei Peligni (JN: 2017); Pescocostanzo, Bosco di S. Antonio (N19; JN: 2018); below Villaggio Mirastelle (JN: 2018). – From the colline (650 m: JN) to the montane (1350 m: JN) belt. On bark of *Acer campestre* (N19), *Acer pseudoplatanus* (N19), *Fagus* (N19; JN), *Quercus cerris* (N19) and *Quercus pubescens* (JN).

***Physcia adscendens* H. Olivier**

Lama dei Peligni (JN: 2017); Monti Pizzi near S. Domenico (JN: 2017); Piano Cerreto near Campo di Giove (JN: 2018); Pescocostanzo, Bosco di S. Antonio (N19; JN: 2018); Valle di Mario (JN: 2018); along the highway Strada Statale 164 (JN: 2018). – From the colline (650 m: JN) to the montane (1434 m: JN) belt. On bark of *Acer campestre* (N19), *Acer pseudoplatanus* (N19; JN), *Fagus* (N19; JN), *Quercus cerris* (JN) and *Ulmus minor* (JN).

***Physcia aipolia* (Humb.) Fürnr.**

Roccacaramanico (NT99; TSB: 1996); Lama dei Peligni (JN: 2017); Monti Pizzi near S. Domenico (JN: 2017); Piano Cerreto near Campo di Giove (JN: 2018); Pescocostanzo, Bosco di S. Antonio (N19; JN: 2018); Valle di Mario (JN: 2018); along the highway Strada Statale 164 (JN: 2018). – From the colline (650 m: JN) to the montane (1434 m: JN) belt. On bark of *Acer campestre* (N19), *Acer pseudoplatanus* (JN), *Fagus* (N19; JN), *Quercus cerris* (JN) and *Ulmus minor* (JN).

**Physcia biziana (A. Massal.) Zahlbr. var. biziana**

Roccacaramanico (NT99; TSB: 1996); Lama dei Peligni (JN: 2017). – From the colline (650 m: JN) to the montane (1000 m: NT99; TSB) belt. On bark of *Ulmus minor* (JN).

***Physcia dubia* (Hoffm.) Lettau**

Roccacaramanico (NT99; TSB: 1996). – In the montane belt (1000 m: NT99; TSB). On calcareous rock (TSB).

***Physcia leptalea* (Ach.) DC.**

Roccacaramanico (NT; TSB: 1996); Pretoro, Colle dell’Angelo (NT99; TSB: 1996); Lama dei Peligni (JN: 2017); Piano Cerreto near Campo di Giove (JN: 2018). – From the colline (650 m: JN) to the montane (1200 m: NT99; TSB) belt. On bark of *Fagus* (TSB), *Quercus cerris* (JN) and *Ulmus minor* (JN).

***Physcia stellaris* (L.) Nyl.**

Majella (C73); Pretoro, Colle dell’Angelo (TSB: 1996); Piano Cerreto near Campo di Giove (JN: 2018); along the highway Strada Statale 164 (JN: 2018). – In the montane belt (1200–1420 m: NT99; TSB; JN). On bark of *Fagus* (TSB; JN) and *Quercus cerris* (JN).

***Physcia tenella* (Scop.) DC.**

Caramanico S. Tommaso (TSB: 1990). – In the colline belt (468 m: TSB). On bark of *Quercus* sp. (TSB).

***Physconia detersa* (Nyl.) Poelt**

Pescocostanzo, Bosco di S. Antonio (N19; JN: 2018). – In the montane belt (1350 m: JN). On bark of *Fagus* (N19; JN).

***Physconia distorta* (With.) J.R. Laundon**

Majella (C73); Roccacaramanico (NT99; TSB: 1996); Lama dei Peligni (JN: 2017); Piano Cerreto near Campo di Giove (JN: 2018); Valle di Mario (JN: 2018); along the highway Strada Statale 164 (JN: 2018); Pescocostanzo, Bosco di S. Antonio (N19). – From the colline (650 m: NT99; TSB) to the montane (1420 m: JN) belt. On bark of *Acer campestre* (N19), *Acer pseudoplatanus* (N19; JN), *Fagus* (N19; JN), *Quercus cerris* (JN) and *Ulmus minor* (JN).

***Physconia enteroxantha* (Nyl.) Poelt**

Monti Pizzi near S. Domenico (JN: 2017); Pescocostanzo, Bosco di S. Antonio (N19; JN: 2018). – In the montane belt (1350–1434 m: JN). On bark of *Acer campestre* (N19) and *Fagus* (N19; JN).

**Physconia muscigena (Ach.) Poelt var. muscigena**

M. Focalone near Bivacco Fusco (TSB: 1996; JN: 2016); Anticima M. Acquaviva (JN: 2016). – In the alpine belt (2490–2600 m: JN). On soil (TSB; JN).

***Physconia perisidiosa* (Erichsen) Moberg**

Caramanico, S. Tommaso (TSB: 1990); Valico della Forchetta (TSB: 1996); Lama dei Peligni (JN: 2017); Pescocostanzo, Bosco di S. Antonio (N19; JN: 2018). – From the colline (468 m: TSB) to the montane (1360 m: JN) belt. On bark of *Acer campestre* (N19; JN), *Fagus* (N19; TSB; JN), deciduous *Quercus* sp. (TSB) and *Ulmus minor* (JN).

***Physconia servitii* (Nádv.) Poelt**

S. Tommaso (TSB: 1990). – In the colline belt (580 m: TSB). On bark of deciduous *Quercus* sp. (TSB).

***Physconia venusta* (Ach.) Poelt**

S. Tommaso (TSB: 1990); Valico della Forchetta (TSB: 1996); Lama dei Peligni (JN: 2017); Pescocostanzo, Bosco di S. Antonio (N19; JN: 2018). – From the colline (580 m: TSB) to the montane (1360 m: JN) belt. On bark of *Fagus* (N19; TSB; JN), *Quercus cerris* (N19) and deciduous *Quercus* sp. (TSB).

***Placidium lachneum* (Ach.) B. de Lesd.**

At six sites along the main ridge of Majella between 2018 and 2620 m (JN: 2019). – From the subalpine (2018 m: JN) to the alpine (2620 m: JN) belt. In high-altitude open habitats (JN). On soil (JN). – The only other records of this taxon from Abruzzo are from the Gran Sasso massif (Nimis and Tretiach 1999). Those from Majella are the southernmost records for Italy (Nimis 2016).

***Placidium squamulosum* (Ach.) Breuss**

Anticima Femmina Morta (JN: 2017); near Campo di Giove (JN: 2018); at 12 sites along the main ridge of Majella between 1812 and 2640 m (JN: 2018, 2019). – From the montane (1250 m: JN) to the alpine (2640 m: JN) belt. In dry grasslands (JN) and high-altitude open habitats (JN). On soil (JN).

***Placocarpus schaereri* (Fr.) Breuss**

Roccacaramanico (NT99; TSB: 1996). – In the montane belt (1000 m: NT99; TSB). On calcareous rock (TSB). – A lichenicolous lichen occurring on *Protoparmeliopsis versicolor*.

***Placopyrenium canellum* (Nyl.) Gueidan & Cl. Roux**

Roccacaramanico (NT99). – In the montane belt (1000 m: NT99). – A lichenicolous lichen occurring on *Circinaria calcarea*.

***Placopyrenium fuscellum* (Turner) Gueidan & Cl. Roux**

Roccacaramanico (NT99; TSB: 1996); hermitage of M. Morrone (NT99; TSB: 1997). – From the colline (500 m: NT99; TSB) to the montane (1000 m: NT99; TSB) belt. On calcareous rock (TSB).

***Placynthiella icmalea* (Ach.) Coppins & P. James**

Pescocostanzo, Bosco di S. Antonio (N19; JN: 2016); at one site along the main ridge of Majella (JN: 2018). – From the montane (1350: JN) to the alpine (2595 m: JN) belt. In high-altitude open habitats (JN). On soil (JN), dead wood (JN) and bark of *Fagus* (N19).

***Placynthium nigrum* (Hudson) Gray**

M. d’Ugni (JN: 2017). – In the subalpine belt (1770 m). On calcareous rock (JN).

***Pleurosticta acetabulum* (Neck.) Elix & Lumbsch**

Majella (C73); Bosco di Pacentro (J74); Pretoro, Colle dell’Angelo (NT99; TSB: 1996); Valico della Forchetta (TSB: 1996); Monti Pizzi near S. Domenico (JN: 2017); Pescocostanzo, Bosco di S. Domenico (N19; JN: 2018); Piano Cerreto near Campo di Giove (JN: 2018); Valle di Mario (JN: 2018); along the highway Strada Statale 164 (JN: 2018). – In the montane belt (1200–1434 m: NT99; TSB; JN). On bark of *Acer campestre* (N19), *Acer pseudoplatanus* (N19; JN), *Fagus* (N19; TSB; JN) and *Quercus cerris* (N19; JN).

***Polyblastia albida* Arnold**

M. Blockhaus (C09). – In the subalpine belt (2170 m: C09). In a pasture (C09). On calcareous rock (C09).

***Polyblastia dermatodes* A. Massal.**

Majelletta (C09); M. Blockhaus (C09); Lettomanoppello (C09). – From the colline (750 m: C09) to the subalpine (2170 m: C09) belt. In open shrublands (C09) and pastures (C09). On calcareous rock (C09). – These records from Majella are the only ones for Abruzzo and peninsular Italy and the southernmost in Italy (Nimis 2016).

***Polyblastia nidulans* (Stenh.) Arnold**

Grotte di Celano near M. Blockhaus (NT99; TSB: 1996); Majelletta (C09). – In the subalpine belt (1850–2150 m: NT99; C09; TSB). On calcareous rock (C09; TSB). – Those from the Majella massif are the only records from Abruzzo (Nimis 2016).

***Polyblastia sendtneri* Kremp.**

Grotte di Celano near M. Blockhaus (NT99; TSB: 1996); above Bivacco Fusco (JN: 2016). – From the subalpine (2150 m: NT99; TSB) to the alpine (2490 m: JN) belt. On calcareous soil (TSB; JN). – Those from the Majella massif are the southernmost records in Italy (Nimis 2016).

***Polyblastia sepulta* A. Massal.**

Pretoro, Colle dell’Angelo (NT99; TSB: 1996); Grotte di Celano near M. Blockhaus (NT99; TSB: 1996); Passo S. Leonardo (C09); Lettomanoppello (C09); Passo Lanciano (C09). – From the colline (750 m: C09) to the subalpine (2150 m: NT99; TSB) belt. In open shrublands (C09) and pastures (C09). On calcareous rock (C09; TSB). – Records from Majella are the only ones from Abruzzo (Nimis 2016).

***Polyblastia verrucosa* (Ach.) Lönnr.**

Grotte di Celano near M. Blockhaus (NT99; TSB: 1996). – In the subalpine belt (2150 m: NT99; TSB). On calcareous rock (TSB). – Records from Majella are the only one for Abruzzo, Apennines and peninsular Italy and the southernmost in Italy (Nimis 2016).

***Polycauliona polycarpa* (Hoffm.) Frödén, Arup & Søchting**

Pretoro, Colle dell’Angelo (NT99; TSB: 1996). – In the montane belt (1200 m: NT99; TSB). On bark of broad-leaved trees (TSB).

***Polysporina urceolata* (Anzi) Brodo**

M. Focalone near Bivacco Fusco (NT99; TSB: 1996); Anticima M. Acquaviva (JN: 2017); M. Macellaro (JN: 2018). – In the alpine belt (2500–2700 m: NT99; TSB; JN). On calcareous rock (TSB; JN).

***Porina oleriana* (A. Massal.) Lettau**

Below the Majelletta (T15; TSB: 2005). – In the montane belt (1350 m: T15; TSB). In a beech forest (T15; TSB). On limestone (T15; TSB). – These are the only known records for Abruzzo (Nimis 2016).

***Porpidia cinereoatra* (Ach.) Hertel & Knoph**

Majella (C73). – This is a silicicolous lichen (Nimis 2016) that likely meets its substrate requirements in the Majella massif on flint limestone.

***Protoblastenia cyclospora* (Körb.) Poelt**

Roccacaramanico (NT99; TSB: 1996). – In the montane belt (1000 m: NT99; TSB). On calcareous rock (TSB).

**Protoblastenia incrustans (DC.) J. Steiner var. incrustans**

Roccacaramanico (NT99; TSB: 1996); Grotte di Celano near M. Blockahus (NT99; TSB: 1996); Anticima Femmina Morta (JN: 2017); M. Macellaro (JN: 2018). – From the montane (1000 m: NT99; TSB) to the alpine (2635 m: JN) belt. On calcareous rock (TSB; JN).

***Protoblastenia rupestris* (Scop.) J. Steiner**

Roccacaramanico (NT99; TSB: 1996); Pretoro, Colle dell’Angelo (NT99; TSB: 1996; S: 1996). – In the montane belt (1000–1200 m: NT99; TSB). On calcareous rock (TSB).

***Protoparmeliopsis admontensis* (Zahlbr.) Hafellner**

M. d’Ugni (JN: 2017). – In the subalpine belt (1770 m: JN). On calcareous rock (JN). – The only other records of this taxon from Abruzzo come from the Gran Sasso massif (Poelt 1958; Poelt and Leuckert 1976).

***Protoparmeliopsis versicolor* (Pers.) M. Choisy**

Roccacaramanico (NT99; TSB: 1996); Anticima Femmina Morta (JN: 2017). – From the montane (1000 m: NT99; TSB) to the alpine (2420 m: JN) belt. On calcareous rock (TSB; JN).

**Pseudevernia furfuracea (L.) Zopf var. furfuracea**

Majella (C73); Bosco di Pacentro (J74); Popoli (TSB: 1986); Macchialunga (JN: 2017). – In the montane belt (1100–1249 m: TSB; JN). On bark of *Fagus* (JN).

***Pseudosagedia aenea* (Körb.) Hafellner & Kalb**

Pretoro, Colle dell’Angelo (NT99; TSB: 1996); Val di Foro (JN: 2018). – In the montane belt (1200 m: NT99; TSB; JN). On bark of *Fagus* (NT99; TSB; JN).

***Psora decipiens* (Hedw.) Hoffm.**

Majella (C73); Majellone (J74); near Bivacco Fusco (JN: 2016); between Grotta Canosa and M. Amaro (JN: 2017); at two sites along the main ridge of Majella between 2560 and 2620 m (JN: 2019). – In the alpine belt (2290–2622 m: JN). In high-altitude open habitats (JN). On soil (JN).

***Punctelia borreri* (Sm.) Krog**

M. Morrone, Osservanza (RV96). – In the colline belt (335 m: RV96). On bark of *Quercus pubescens* (RV96).

***Pyrenodesmia albopruinosa* (Arnold) S.Y. Kondr.**

Roccacaramanico (NT99; TSB: 1996); M. Focalone near Bivacco Fusco (NT99; TSB: 1996). – From the montane (1000 m: NT99; TSB) to the alpine (2500 m: NT99; TSB) belt. On calcareous rock (TSB).

***Pyrenodesmia alociza* (A. Massal.) Arnold**

Roccacaramanico (NT99; TSB: 1996); Anticima M. Acquaviva (JN: 2016); Anticima Femmina Morta (JN: 2017); M. Macellaro (JN: 2018). – From the montane (1000 m: NT99; TSB) to the alpine (2635 m: JN) belt. On calcareous rock (TSB; JN).

***Pyrenodesmia chalybaea* (Fr.) A. Massal.**

Roccacaramanico (NT99; TSB: 1996); Eremo di M. Morrone (NT99; TSB: 1997); between Lettomanoppello and Passo Lanciano (TSB: 2005). – From the colline (500 m: NT99; TSB) to the montane (1080 m: TSB) belt. On calcareous rock (TSB).

***Pyrenodesmia erodens* (Tretiach, Pinna & Grube) Søchting, Arup & Frödén**

Anticima M. Acquaviva (JN: 2016); Anticima Femmina Morta (JN: 2017). – In the alpine belt (2420–2600 m: JN). On calcareous rock (JN).

***Pyrenodesmia variabilis* (Pers.) A. Massal.**

Hermitage of M. Morrone (NT99; TSB: 1997); near Martellose (JN: 2017). – From the colline (500 m: NT99; TSB) to the alpine (2065 m: JN) belt. On calcareous rock (TSB; JN).

***Pyrenula nitida* (Weigel) Ach.**

Valle di Mario (JN: 2018); Val di Foro (JN: 2018); below Villaggio Mirastelle (JN: 2018). – In the montane belt (1200–1230 m: JN). On bark of *Fagus* (JN).

***Ramalina farinacea* (L.) Ach.**

Majella (J74); Monti Pizzi near S. Domenico (JN: 2017); Valle di Mario (JN: 2018); Piano Cerreto near Campo di Giove (JN: 2018); along the highway Strada Statale 164 (JN: 2018). – In the montane belt (1420–1440 m: JN). On bark of *Acer pseudoplatanus* (JN), *Fagus* (JN) and *Quercus cerris* (JN).

***Ramalina fastigiata* (Pers.) Ach.**

Popoli (TSB: 1986); Monti Pizzi near S. Domenico (JN: 2017); Pescocostanzo, Bosco di S. Antonio (N19; JN: 2018). – In the montane belt (1350–1440 m). On bark of *Fagus* (N19; JN).

***Ramalina fraxinea* (L.) Ach.**

Majella (C73; J74); Popoli (TSB: 1986); Pretoro, Colle dell’Angelo (NT99; TSB: 1996); Valico della Forchetta (TSB: 1996); Monti Pizzi near S. Domenico (JN: 2017); Palena, Fontana delle Rose (JN: 2018); Cansano (JN: 2018); Pescocostanzo, Bosco di S. Antonio (N19; JN: 2018); Valle di Mario (JN: 2018); along the highway Strada Statale 164 (JN: 2018). – In the montane belt (1100–1440 m: JN). On bark of *Acer pseudoplatanus* (JN), *Fagus* (N19; JN) and *Quercus cerris* (N19).

***Ramonia luteola* Vězda**

Sella di Grotta Canosa (JN: 2018). – In the montane belt (1200 m: JN). On bark of *Fagus* (JN). – First record for Abruzzo and southernmost record in Italy (cf. Nimis 2016). The species is included in the Italian Red List of epiphytic lichens as “vulnerable” (Nascimbene et al. 2013).

***Rhizocarpon atroflavescens* Lynge**

Grotte di Celano (NT99; TSB: 1996). – In the subalpine belt (2150 m: NT99; TSB). On calcareous rock (TSB). – This is the only record for Abruzzo and peninsular Italy and the southernmost one in Europe (Nimis 2016). This is a slightly silicicolous lichen (Nimis 2016) that meets its substrate requirements in the Majella massif on flint limestone.

***Rhizocarpon badioatrum* (Spreng.) Th. Fr.**

Majella (C73); Valle dell’Orfento (J74). – This is a silicicolous lichen (Nimis 2016) that likely meets its substrate requirements in the Majella massif on flint limestone.

***Rhizocarpon umbilicatum* (Ramond) Flagey**

Majella (C73); Valle dell’Orfento (J74); trail between Blockhaus and M. Focalone (TSB: 2005). – In the alpine belt (2300 m: TSB). On calcareous rock (TSB).

***Rinodina bischoffii* (Hepp) A. Massal.**

Roccacaramanico (NT99; TSB: 1996); hermitage of M. Morrone (NT99; TSB: 1997). – From the colline (500 m: NT99; TSB) to the montane (1000 m: NT99; TSB) belt. On calcareous rock (TSB).

***Rinodina guzzinii* Jatta**

Roccacaramanico (NT99; TSB: 1996). – In the montane belt (1000 m: NT99; TSB). On calcareous rock (TSB).

***Rinodina immersa* (Körb.) J. Steiner**

Roccacaramanico (NT99; TSB: 1996); Passo San Leonardo (C09); Majelletta (C09); Anticima M. Acquaviva (JN: 2017); M. Macellaro (JN: 2018). – From the montane (1000 m: NT99; TSB) to the alpine (2635 m: JN) belt. On calcareous rock (TSB; JN).

***Rinodina lecanorina* (A. Massal.) A. Massal.**

Roccacaramanico (NT99; TSB: 1996). – In the montane belt (1000 m: NT99; TSB). On calcareous rock (TSB).

***Rinodina roscida* (Sommerf.) Arnold**

At ten sites along the main ridge of Majella between 2250 and 2664 m (JN: 2018, 2019). – From the subalpine (2250 m: JN) to the alpine (2664 m: JN) belt. In high-altitude open habitats (JN). On bryophytes and plant debris (JN). – New to Abruzzo. These are the only records for Apennines and peninsular Italy and the southernmost in Italy (Nimis 2016).

***Rinodina sophodes* (Ach.) A. Massal.**

Roccacaramanico (NT99; TSB: 1996); Pretoro, Colle dell’Angelo (NT99; TSB: 1996); hermitage of M. Morrone (NT99; TSB: 1997); Pescocostanzo, Bosco di S. Antonio (N19; JN: 2018). – From the colline (500 m: NT99; TSB) to the montane (1350 m: JN) belt. On bark of *Fagus* (N19; TSB; JN) and *Fraxinus ornus* (TSB).

***Romjularia lurida* (Ach.) Timdal**

Majella (C73); Majellone (J74); Roccacaramanico (NT99; TSB: 1996); at one site along the main ridge of Majella (JN: 2019). – From the montane (1000 m: NT99; TSB) to the subalpine (1958 m: JN) belt. In high-altitude open habitats (JN). On calcareous soil (TSB; JN).

***Rostania ceranisca* (Nyl.) Otálora, P.M. Jørg. & Wedin**

At one site along the main ridge of Majella (JN: 2019). – In the alpine belt (2662 m: JN). In high-altitude open habitats (JN). On soil (JN). – New to Abruzzo. This is the only record for Apennines and peninsular Italy and the southernmost in Italy (Nimis 2016).

**Rusavskia elegans (Link) S.Y. Kondr. & Kärnefelt subsp. elegans**

Femmina Morta (J74; JN: 2017); Roccacaramanico (NT99; TSB: 1996); M. Focalone near Bivacco Fusco (NT99; TSB: 1996); Anticima M. Acquaviva (JN: 2017); Sella di Grotta Canosa (JN: 2017); at one site along the main ridge of Majella (JN: 2018). – From the montane (1000 m: NT99; TSB) to the alpine (2669 m: JN) belt. In high-altitude open habitats (JN). On calcareous rock (TSB; JN).

***Rusavskia sorediata* (Vain.) S.Y. Kondr. & Kärnefelt**

M. Focalone near Bivacco Fusco (NT99; TSB: 1996); Grotta Canosa (JN: 2017). – In the alpine belt (2500–2559 m: TSB; JN). On calcareous rock (TSB; JN).

***Sarcogyne hypophaea* (Nyl.) Arnold**

Roccacaramanico (NT99; TSB: 1996). – In the montane belt (1000 m: NT99; TSB). On calcareous rock (TSB).

**Sarcogyne regularis Körb. var. regularis**

Hermitage of M. Morrone (NT99; TSB: 1997). – In the colline belt (500 m: NT99; TSB). On calcareous rock (TSB).

***Sclerophora pallida* (Pers.) Y.J. Yao & Spooner**

Pescocostanzo, Bosco di S. Antonio (N19; JN: 2018). – In the montane belt (1350 m: JN). On bark of *Acer campestre* (N19; JN) and *Fagus* (N19; JN). – The species is included in the Italian Red List of epiphytic lichens as “vulnerable” (Nascimbene et al. 2013).

**Scoliciosporum umbrinum (Ach.) Arnold var. corticicolum**

Pretoro, Colle dell’Angelo (NT99; TSB: 1996); Valico della Forchetta (TSB: 1996). – In the montane belt (1200–1360 m: NT99; TSB). On bark of *Fagus* (TSB).

This name, whose taxonomic value is uncertain, is applied to corticolous populations of a *Scoliciosporum* with the hymenial characters of *S. umbrinum* (Nimis et al. 2018). In Nimis (2016) this taxon is not reported and therefore it is formally new to Italy.

***Scytinium gelatinosum* (With.) Otálora, P.M. Jørg. & Wedin**

Hermitage of M. Morrone (NT99; TSB: 1997); Val di Foro (JN: 2018). – From the colline (500 m: NT99; TSB) to the montane (1250 m: JN) belt. On calcareous soil (TSB) and terricolous mosses (JN).

***Scytinium imbricatum* (P.M. Jørg.) Otálora, P.M. Jørg. & Wedin**

Near Campo di Giove (JN: 2018); at 23 sites along the main ridge of Majella between 1990 and 2664 m (JN: 2018, 2019). – From the montane (1250 m: JN) to the alpine (2664 m: JN) belt. In dry grasslands (JN) and high-altitude open habitats (JN). On soil (JN). – New to Abruzzo. These are the only records for Apennines and peninsular Italy and the southernmost in Italy (Nimis 2016).

***Scytinium lichenoides* (L.) Otálora, P.M. Jørg. & Wedin**

Majella (C73; J74); Roccacaramanico (NT99; TSB: 1996); Pescocostanzo, Bosco di S. Antonio (N19; JN: 2016, 2018); Lama dei Peligni (JN: 2017); Val di Foro (JN: 2018); Centiata, Villaggio Mirastelle (JN: 2018). – From the colline (650 m: JN) to the montane (1350 m: JN) belt. On bark of *Fagus* (N19; JN) and calcareous soil (TSB).

***Scytinium schraderi* (Ach.) Otálora, P.M. Jørg. & Wedin**

At four sites along the main ridge of Majella (JN: 2019). – From the subalpine (2081 m: JN) to the alpine (2300 m: JN) belt. In high-altitude open habitats (JN). On soil (JN).

***Seirophora contortuplicata* (Ach.) Frödén**

Grotte di Celano near M. Blockhaus (NT99; TSB: 1996); Anticima Femmina Morta (JN: 2017). – From the subalpine (2150 m: NT99; TSB) to the alpine (2420 m: JN) belt. On calcareous rock (TSB; JN).

**Solorina bispora Nyl. subsp. bispora**

Anticima M. Acquaviva (JN: 2016). – In the alpine belt (2600 m: JN). On soil (JN).

**Solorina bispora subsp. macrospora (Harm.) Burgaz & I. Martínez**

Near Bivacco Fusco (JN: 2016); at one site along the main ridge of Majella (JN: 2019). – In the subalpine belt (2230–2290 m: JN). In high-altitude open habitats (JN). On soil (JN). – New to Abruzzo. These are the only records for Apennines and peninsular Italy and the southernmost in Italy (Nimis 2016).

**Squamarina cartilaginea (With.) P. James var. cartilaginea**

Majella (C73; J74); hermitage of M. Morrone (NT99; TSB: 1997); Valle di Fara (JN: 2017); trail between Lama dei Peligni and Rifugio Fonte Tarì (JN: 2017). – From the colline (500 m: NT99; TSB) to the montane (1135 m: JN) belt. On calcareous soil (TSB) and rock (JN).

***Squamarina gypsacea* (Sm.) Poelt**

Near Martellose (JN: 2017). – In the subalpine belt (2065 m: JN). On calcareous rock (JN).

***Squamarina lentigera* (Weber) Poelt**

Campo di Giove (JN: 2018); near Campo di Giove (JN: 2018). – In the montane belt (1200–1250 m: JN). In dry grasslands (JN). On soil (JN).

***Squamarina stella-petraea* Poelt**

Roccacaramanico (NT99; TSB: 1996); hermitage of M. Morrone (NT99; TSB: 1997). – From the colline (500 m: NT99; TSB) to the montane (1000 m: NT99; TSB) belt. On calcareous rock (TSB) and soil (TSB).

***Staurothele orbicularis* (A. Massal.) Th. Fr.**

Caramanico (C09). – In the colline belt (570 m: C09). In a pasture with scattered shrubs (C09). On calcareous rock (C09).

***Synalissa ramulosa* (Bernh.) Fr.**

Roccacaramanico (NT99; TSB: 1996). – In the montane belt (1000 m: NT99; TSB). On calcareous rock (TSB).

***Tephromela atra* (*Huds* .) *Hafellner* var. torulosa (Flot.) Hafellner**

Monti Pizzi near S. Domenico (JN: 2017). – In the montane belt (1434 m: JN). On bark of *Fagus* (JN).

***Tetramelas geophilus* (Sommerf.) Norman**

Majella (C73). – The historical record was not confirmed recently, but is considered as reliable, since this is a widespread species (Nimis 2016).

***Thalloidima candidum* (Weber) A. Massal.**

Majella (C73; J74); Grotte di Celano near M. Blockhaus (NT99). – In the subalpine belt (2150 m: NT99).

***Thalloidima diffractum* (A. Massal.) A. Massal.**

Grotte di Celano near M. Blockhaus (TSB: 1996); near Bivacco Fusco (JN: 2016); Sella di Grotta Canosa (JN: 2017); Rava della Vespa (JN: 2017). – From the subalpine (2150 m: TSB) to the alpine (2643 m: JN) belt. On calcareous rock (TSB; JN) and soil (JN).

***Thalloidima sedifolium* (Scop.) Kistenich, Timdal, Bendiksby & S. Ekman**

Majella (C73; J74); near Bivacco Fusco (JN: 2016); Cima dell’Altare (JN: 2017); Campo di Giove (JN: 2018); near Campo di Giove (JN: 2018); at one site along the main ridge of Majella (JN: 2019). – From the montane (1250 m: JN) to the alpine (2490 m: JN) belt. In dry grasslands (JN) and high-altitude open habitats (JN). On calcareous soil (JN).

***Thelidium decipiens* (Nyl.) Kremp.**

Anticima M. Acquaviva (JN: 2017); M. Macellaro (JN: 2018). – In the alpine belt (2635–2700 m: JN). On calcareous rock (JN).

***Thelidium dionantense* (Hue) Zschacke**

Grotte di Celano near M. Blockhaus (NT99; TSB: 1996). – In the subalpine belt (2150 m: NT99; TSB). On calcareous rock (TSB). – This is the only known record from Italy (Nimis 2016).

***Thelidium incavatum* Mudd**

M. Focalone (C09). – In the alpine belt (2600 m: C09). On calcareous rock (C09). – This is the only record from Abruzzo (Nimis 2016).

***Thelidium papulare* (Fr.) Arnold**

Pretoro, Colle dell’Angelo (NT99; TSB: 1996); Anticima M. Acquaviva (JN: 2017). – From the montane (1200 m: NT99; TSB) to the alpine (2700 m: JN) belt. On calcareous rock (TSB; JN).

***Toninia subnitida* (Hellb.) Hafellner & Türk**

Anticima Femmina Morta (JN: 2017). – In the alpine belt (2420 m: JN). On rock (JN). – New to Abruzzo. This is the only record for central Italy (Nimis 2016).

***Toniniopsis coelestina* (Anzi) Kistenich, Timdal, Bendiksby & S. Ekman**

Below Bivacco Fusco (JN: 2016). – In the subalpine belt (2290 m: JN). On soil (JN). – New to Abruzzo. This is the only record for peninsular Italy and the Apennines and the southernmost record in Italy (Nimis 2016).

***Trapeliopsis gelatinosa* (Flörke) Coppins & P. James**

At two sites along the main ridge of Majella between 2579 and 2634 m (JN: 2019). – In the alpine belt (2579–2634 m: JN). In high-altitude open habitats (JN). On soil (JN). – New to Abruzzo. This is the southernmost record in Italy (Nimis 2016).

***Umbilicaria cylindrica* (L.) Delise**

Majella (J74). – This is a silicicolous lichen (Nimis 2016) that likely meets its substrate requirements in the Majella massif on flint limestone. This is the only record from Abruzzo (Nimis 2016).

***Usnea barbata* (L.) F.H. Wigg.**

Majella (C73). – Most Italian records of the genus *Usnea* would require accurate revision and this historical record was not confirmed recently. However, we considered it as reliable, since the ecological conditions required by this species (Nimis 2016) occur within the study area.

***Usnea dasopoga* (Ach.) Nyl.**

Bosco di Pacentro (J74). – Most Italian records of the genus *Usnea* would require accurate revision, and this historical record was not confirmed recently. However, we considered it as reliable, since the ecological conditions required by this species (Nimis 2016) occur within the study area.

***Variospora aurantia* (Pers.) Arup, Frödén & Søchting**

Majella (C73). – The historical record was not confirmed recently, but is considered as reliable, since this is a widespread species (Nimis 2016).

***Variospora velana* (A. Massal.) Arup, Søchting & Frödén**

Roccacaramanico (NT99; TSB: 1996); hermitage of M. Morrone (NT99; TSB: 1997). – From the colline (500 m: NT99; TSB) to the montane (1000 m: NT99; TSB) belt. On calcareous rock (TSB).

***Verrucaria hochstetteri* Fr.**

M. Focalone (C09); M. d’Ugni (JN: 2017); M. Macellaro (JN: 2018). – From the subalpine (1770 m: JN) to the alpine (2635 m: JN) belt. On calcareous rock (C09; JN).

**Verrucaria nigrescens Pers. f. nigrescens**

Pretoro, Colle dell’Angelo (NT99; TSB: 1996); hermitage of M. Morrone (NT99; TSB: 1997). – From the colline (500 m: NT99; TSB) to the montane (1200 m: NT99; TSB) belt. On calcareous rock (TSB).

***Verrucula biatorinaria* (Zehetl.) Nav.-Ros. & Cl. Roux**

Grotte di Celano near M. Blockhaus (NT99; TSB: 1996). – In the subalpine belt (2150 m: NT99; TSB). On calcareous rock (TSB). – A lichenicolous lichen occurring on *Calogaya biatorina*.

***Verrucula coccinearia* (Zehetl.) Nav.-Ros. & Cl. Roux**

Grotte di Celano near M. Blockhaus (NT99; TSB: 1996); Anticima Femmina Morta (JN: 2017). – From the subalpine (2150 m: NT99; TSB) to the alpine (2420 m: JN) belt. On calcareous rock (TSB; JN). – A lichenicolous lichen occurring on *Caloplaca coccinea*. Those from Majella are the only records for Abruzzo, Apennines and peninsular Italy and the southernmost in Italy (Nimis 2016).

***Verrucula granulosaria* (Clauzade & Zehetl.) Nav.-Ros. & Cl. Roux**

Roccacaramanico (NT99). – In the montane belt (1000 m: NT99). – This is a lichenicolous lichen occurring on *Flavoplaca granulosa*. In Nimis and Tretiach (1999), it was reported under *Verrucula latericola*.

***Vulpicida pinastri* (Scop.) J.-E. Mattsson & M.J. Lai**

Majella (C73); M. Amaro (J74). – On bark of *Pinus* sp. (J74). – The historical records were not confirmed recently, but are considered as reliable, since the ecological conditions required by this species (Nimis 2016) occur within the study area.

***Xanthocarpia lactea* (A. Massal.) A. Massal.**

Hermitage of M. Morrone (NT99; TSB: 1997); Anticima M. Acquaviva (JN: 2017); M. Macellaro (JN: 2018). – From the colline (500 m: NT99; TSB) to the alpine (2700 m: JN) belt. On calcareous rock (TSB; JN).

***Xanthocarpia marmorata* auct.**

Hermitage of M. Morrone (NT99; TSB: 1997). – In the colline belt (500 m: NT99; TSB). On calcareous rock (TSB).

***Xanthoria parietina* (L.) Th. Fr.**

Majella (C73; J74); Roccacaramanico (NT99; TSB: 1996); Pretoro, Colle dell’Angelo (NT99; TSB: 1996); Lama dei Peligni (JN: 2017); Pescocostanzo, Bosco di S. Antonio (N19; JN: 2018); Piano Cerreto near Campo di Giove (JN: 2018); Valle di Mario (JN: 2018); along the highway Strada Statale 164 (JN: 2018). – From the colline (650 m: JN) to the montane (1420 m: JN) belt. On bark of *Acer campestre* (N19), *Acer pseudoplatanus* (N19; JN), *Fagus* (N19; TSB; JN), *Quercus cerris* (JN) and *Ulmus minor* (JN).

### Dubious records

***Caloplaca subochracea* auct.**

Femmina Morta (J74). – This is a mainly coastal species (Nimis 2016) whose occurrence in the study area is dubious.

***Cladonia scabriuscula* (Delise) Nyl.**

Majella (C73). – This is a rare species in Italy and is not reported by other sources in central-southern Apennines. The record would require confirmation.

***Dermatocarpon complicatum* (Lightf.) W. Mann**

Majella (C73); Femmina Morta (J74). – This is a critical taxon mainly growing on periodically inundated siliceous rocks (Nimis 2016) and it would need to be confirmed in the study area. This would be the only record for Abruzzo.

***Leptogium brebissonii* Mont.**

Valle dell’Orfento (RV96). – In the colline belt (570 m: RV96). On sandstone (RV96). This species is usually epiphytic (Nimis 2016) and therefore this record would require confirmation.

***Lichina confinis* (O.F. Müll.) C. Agardh**

Majella (C73); Valle dell’Orfento (J74). – This is a coastal species occurring on rocks at the interface between the littoral and the mesic supralittoral belts (Nimis 2016) whose occurrence in the study area is very dubious. The record was reported by Jatta under the name *Lichina elisabethae* A. Massal.

***Lobaria linita* (Ach.) Rabenh.**

Majella (C73); Bosco di Pacentro (J74). – This record reported by Jatta was collected “ad terram inter muscos in sylva Pacentri”. According to Nimis (1993), old records from central-southern Italy should be referred to *L. pulmonaria*, since *L. linita* is restricted to the Alps in Italy.

***Rinodina oxydata* (A. Massal.) A. Massal.**

Valle dell’Orfento (J74). – This is a silicicolus taxon whose occurrence in the Majella massif would be related to flint limestoine. Since this would be the only record for Abruzzo, it requires confirmation.

## Discussion

This checklist provides a baseline of the lichens known to occur in the Majella National Park, highlighting the potential of this area as a hotspot of lichen biodiversity, especially from a biogeographical point of view. On one hand, the high number of regionally-new taxa discovered during our recent investigations suggests that further research is needed to reach a more exhaustive picture of the lichen biota of Abruzzo, as well as of the Majella massif. In particular, a more intensive collection in rocky and forest habitats, as well as in high elevation ranges, is likely to produce a relevant increase in the number of species.

On the other hand, the occurence of many arctic-alpine taxa (see Nimis 1997; Nimis and Tretiach 1995) that reach here their southernmost Italian or European distribution limit and the occurence of steppic chorotypes, as in the case of *Circinaria hispida*, confirm the phytogeographical peculiarity of this area also for lichens (see Conti et al. 2019 for vascular plants; Nimis 2016 for lichens). In the core of the Mediterranean Region, small, disjunct populations of artic-alpine taxa that are disjunct from those of the Alps are currently restricted to the highest 200 m of the Majella massif. In a global change perspective, this cold-adapted, disjunct component of the lichen biota is strongly exposed to the impact of warming conditions, as in the emblematic case of *Allocetraria madreporiformis* whose main local populations almost exclusively occur within *Salix retusa* islands in the Macellaro summit. The past establishment and current persistence of these cold-adapted taxa are likely related to the great extension of the high altitude area characterised by vast plateaus that may provide microrefugia (e.g. small-scale cold refugia) suitable for these small-sized organisms (Conti et al. 2019).

In addition, the epiphytic lichen biota is noteworthy, including several species of conservation concern that are Red-listed in Italy (Nascimbene et al. 2013; a national Red List is currently available only for epiphytic species). This indicates that the forests of the Majella National Park effectively contribute to the conservation of endangered epiphytic species of the Italian lichen biota. The best conserved part of the “Bosco di S. Antonio” forest is an emblematic example of this situation, hosting species sensitive to human disturbance as *Lobaria pulmonaria* or rare calicioid lichens (Nascimbene et al. 2019).
